# Which representations of their gender group affect men’s orientation towards care? the case of parental leave-taking intentions

**DOI:** 10.1371/journal.pone.0260950

**Published:** 2021-12-03

**Authors:** Carolin Scheifele, Melanie C. Steffens, Colette Van Laar

**Affiliations:** 1 Center for Social and Cultural Psychology, University of Leuven, Leuven, Belgium; 2 PhD Fellow of the Research Foundation–Flanders, Brussels, Belgium; 3 Department of Social, Environmental, and Economic Psychology, University of Koblenz-Landau, Landau, Germany; Goethe University Frankfurt am Main, GERMANY

## Abstract

Men are currently underrepresented in traditionally female care-oriented (communal) engagement such as taking parental leave, whereas they are overrepresented in traditionally male (agentic) engagement such as breadwinning or leadership. We examined to what extent different prototypical representations of men affect men’s self-reported parental leave-taking intentions and more generally the future they can imagine for themselves with regard to work and care roles (i.e., their possible selves). We expected prototypes of men that combine the two basic stereotype dimensions of agency and communion to increase men’s communal intentions. In two experiments (*N*_1_ = 132, *N*_2_ = 233), we presented male participants with contrived newspaper articles that described the ideal man of today with varying degrees of agency and communion (between-subjects design with four conditions; combined agentic and communal vs. agentic vs. communal vs. control condition). Results of Experiment 1 were in line with the main hypothesis that especially presenting a combination of agency and communion increases men’s expectations for communal engagement: As compared to a control condition, men expected more to engage in caretaking in the future, reported higher parental leave-taking intentions, and tended to expect taking longer parental leave. Experiment 2 only partially replicated these findings, namely for parental leave-taking intentions. Both experiments additionally provided initial evidence for a contrast effect in that an exclusive focus on agency also increased men’s self-reported parental leave-taking intentions compared to the control condition. Yet, exclusively emphasizing communion in prototypes of men did not affect men’s communal intentions, which were high to begin with. We further did not find evidence for preregistered mechanisms. We discuss conditions and explanations for the emergence of these mixed effects as well as implications for the communication of gendered norms and barriers to men’s communal engagement more broadly.

## Introduction

In the last 50 or so years, men’s traditional gender roles have shown fewer changes than women’s. Women have increasingly entered the labor market and attached greater importance to the work domain in their lives. Men’s gender roles have shown much more inertia: Men are not taking on care roles (also named communal roles) to the same degree as women have claimed traditionally male-dominated work roles and leadership positions for themselves [1, 2]. Men’s underrepresentation in communal roles manifests not only in the domestic domain but also in the work sphere. For example, only one-tenth to one-fifth of registered nurses and elementary and middle school teachers are male in the USA and in Germany, where the current studies were conducted [3–5]. In the domestic sphere, men spend about half as much time as women on daily household tasks and childcare [6, 7]. Men’s uptake of parental leave has increased, and 37% of fathers took leave in 2016 in Germany; yet, often for only short periods of up to two months (as compared to the 10 to 12 months that mothers take) [8].

Because of this underrepresentation, men themselves, their partners, their children, and society miss out on various benefits associated with men’s increased engagement in communal roles [1, 2]. From a societal and labor market perspective, motivating such a large group as men to engage in communal roles can help to meet the high demands for professionals in health, child, and geriatric care [9]. On an individual level, communal orientation has been linked to positive psychological outcomes such as increased well-being and relationship satisfaction [10–13]. In dual-earner couples, men’s communal engagement can leave room for their partner’s career pursuits. Therefore, in heterosexual relationships, which the current paper focuses on, men’s communal engagement can improve women’s financial security and pension provision [14]. Regarding parenting, men’s involvement beyond resource provision has been found to be beneficial for their children’s developmental, psychosocial, and educational outcomes [15] and to motivate daughters to consider less female gender-stereotypical occupations [16]. To reap these benefits for men themselves and their proximal family environment, parental leave can represent an effective tool. Even more, men’s parental leave-taking has been associated with their continuing engagement in childcare [17–19], changes in grandparents’ attitudes towards gender equality [20], and in colleagues’ willingness to take parental leave themselves [21].

Prior research has found evidence for a variety of factors that may keep men from taking up more parental leave such as specific leave policies, financial considerations, organizational norms, or couples’ negotiations of paid and unpaid work [22–28]. From a psychological perspective, possible external barriers such as facing stigmatization and job disadvantages have been studied [29–31]. Less attention has been paid to the role of masculinity norms and the extent to which communal traits are integrated in such for men’s leave-taking intentions. Thus, in the present studies we examine how different prototypical representations of men varying in communal (e.g., emotional, empathetic, trustworthy) and agentic (e.g., ambitious, assertive, competent) content affect men’s communal orientation in general and their parental leave-taking intentions more specifically.

### Masculinity norms, fathering norms, and parental leave

When examining how ascribing communal traits to men themselves and their gender group relates to their parental leave-taking intentions, it is important to consider recent changes in masculinities and fathering norms. A theoretical basis for such examinations are discussions about a shift from hegemonic to caring masculinities with regard to caregiving fathers [32]. According to its original formulation, hegemonic masculinity represents the most honored form of masculinity and perpetuates the dominance of men over women [33, 34]. On the contrary, caring masculinities reject this dominance and reconcile masculinity with traditionally feminine characteristics such as nurturance, emotionality, and connection [32, 35]. Many men likely neither enact hegemonic nor caring masculinities in pure form or identify exclusively with such; however, both represent normative standards for men regarding what it means to be a man [32, 34].

In empirical studies, similar shifts from traditional to so-called new, caring norms for men and fathers can be observed. Recent examinations of panel data in the US and Europe show that traditional gender ideologies, associating men with breadwinning and women with caretaking, are declining and that egalitarianism, although in various forms, is on the rise [36–38]. In terms of gender stereotypes, a recent study showed that men perceive other men and especially themselves as actually rather communal (although women still rated themselves as more communal) [39]. Similarly, now more than in the past the “new father” is associated with more maternal (i.e., communal) traits and behaviors, and people expect a further alignment of motherhood and fatherhood for the future [40, also see 41]. In a German study, two thirds of respondents considered breadwinning and nurturing as equally important for an ideal father, while one third even prioritized nurturing over breadwinning [42].

Although men and fathers are increasingly associated with care, these new ideals for manhood and fatherhood have not fully replaced traditional norms and stereotypes. Communion is still firmly associated with women, and stereotypes of men appear more resistant to change than those of women [43–45]. In terms of role engagement, implicit tests showed that parenthood is more strongly associated with women than men [46], and only given role change, stereotype change can eventually be expected [47]. Even though a trend towards neutrality within gender stereotypes can be observed, it is estimated to take at least 134 years for implicit male-career/female-family associations to dissolve [48]. These findings emerged in representative as well as student samples and in different national contexts. Thus, new forms of masculinity and fatherhood, although popular in societal debates, do not seem to have replaced traditional forms [32]. Men, and especially fathers, still need to negotiate with some difficulty their role between being the main provider for their families and being an involved, primary caregiving father. Similar to when women increasingly entered the labor force, men are now facing the pressures of “having it all” [49]. This balancing of modern and traditional masculinities and fathering norms is evident in recent research on parental leave across cultural contexts. On the one hand, case studies and interviews with Swedish families support the emergence of child- and family-oriented masculinity norms which affect men’s parental leave-taking [50, 51]. On the other hand, French couples, contrary to Swedish ones, adhered more strongly to traditional forms of masculinity and did not see men’s leave-taking as an option [23]. In Austria, parental leave was perceived to be in line with masculinity norms; but only if men personally wanted to take leave and external circumstances allowed for it [52]. In cases when men do take leave, especially independently from their partners, they often have to find ways to integrate caretaking into male gender roles, for example, by defining childcare as “hard work” [53, 54].

### Agency and communion in male gender norms and prototypes

As outlined, the relevance of masculinities and fathering norms and the degree to which caretaking and communion have been integrated with traditional norms focusing on breadwinning and agency has been considered by past research. Yet, it remains unclear which norms specifically motivate men to increase their engagement in care roles and how varying descriptions of a prototypical man can affect men’s parental leave-taking intentions. The central hypothesis of the current experiments is that norms describing prototypical men as both agentic *and* communal are most likely to increase men’s communal intentions, for example, regarding parental leave.

Masculinity norms as a subset of gender norms describe what men typically do and what is thus “normal” (*descriptive norms*) as well as what men should or should not do (*injunctive or prescriptive norms*) [55–57]. Within the social identity approach [58, 59], the representation of what an individual perceives as normative for a group is called the group prototype [60]. Prototypes have been defined as “the ideal-type member of a category that best represents its identity in a given context and frame of reference” [61, following 62]. As such, prototypes do not necessarily represent average group members but rather capture the essence of a group, often in ideal or hypothetical form (thus similar to prescriptive norms) [63]. When individuals are perceived through the lens of a prototype (i.e., viewed as group members with similar attributes rather than individuals), we speak of stereotyping [63]. Stereotypes of men are traditionally characterized by agency [57]. Agency, along with communion, represent the two fundamental content dimensions for perceiving the self, others, and social groups [64–68]. These “Big Two” also emerged when examining gender stereotypes and typically male and female attributes [69, 70], and recent theorizing even sees gender as the source of the formation of these fundamental dimensions [71]. Agentic traits and behaviors which are traditionally associated with men include being assertive, independent, competitive, and dominant and taking on respective roles (e.g., leadership). In contrast, women are associated more, and men less, with the second dimension, communion, which includes being friendly, caring, understanding, and emotional, and taking on respective roles such as caretaking [56, 57]. Although these strong associations are blurring to some extent (as described above), parental leave and care-oriented, communal engagement in general is traditionally considered counter-stereotypic for men. When men nevertheless engage in such roles and behaviors, they may encounter backlash (i.e., social and economic sanctions such as being perceived as less masculine and more feminine and receiving worse job evaluations) [for a review see 72].

The role prioritization model [73] suggests a possible solution for avoiding backlash for engaging in counter-stereotypic behavior. According to the model, men (and women) can receive leeway to engage in counter-stereotypic behavior if they are perceived as prioritizing traditionally stereotypic roles and only augmenting, instead of replacing them, with counter-stereotypic behavior. Thus, men who are perceived as prioritizing breadwinning and agentic roles in general should receive fewer sanctions for engaging in communal, caretaking roles [73]. Possibly, such “licensing” is not only beneficial when being evaluated by others but could also give individuals themselves the assurance to act in counter-stereotypic ways when being confronted with masculinity norms. Thus, we argue that not only being perceived as balancing traditionally stereotypic agentic and counter-stereotypic communal aspects could be beneficial for men. In addition, learning that others value both aspects in men could enable men to increase their communal engagement.

First empirical evidence related to these assumptions for masculinity norms has been gained in a study on the effect of different peer norms for young men’s communal outcomes [74, also see 75]. When male participants learned that peers valued agency as well as communion in an ideal man, they showed more communal outcomes compared to a control condition (i.e., had more communal self-concepts, intended to hide communal task engagement less, and had more progressive gender-related attitudes). In addition to being confronted with a combination of agency and communion, male participants also indicated more progressive attitudes towards gender-related social change, when their peers supposedly described the ideal man as entirely agentic [74]. In light of shifting masculinity norms, this strictly traditional notion of what it means to be a man could have motivated some men to indicate holding contrasting beliefs. As masculinity norms are broadening, an exclusive focus on agency could be perceived as extreme, unambiguous, and one-sided. When points of reference are characterized by such attributes, contrast effects can be the result (i.e., being pushed away from the point of reference) [76, 77]. In the study by van Grootel and colleagues [74], we interpret another finding as a contrast effect: When male peers were said to perceive communal traits as most desirable for men, participants intended to hide communal task engagement the most. Thus, ideal-type representations of men seem to either encourage or discourage men to show communal intentions and progressive attitudes depending on presented degrees of agency and communion. Still, past research showed that male gender roles and stereotypes are rather resistant to change [43–45, 78] and that it could be especially difficult for men to consider counter-stereotypic engagement [1, 45]. Therefore, we additionally examine an outcome variable that could be more open to counter-stereotypic content and represents a broader indicator of men’s communal intentions than parental leave-taking intentions: men’s possible selves.

### Men’s possible selves and caretaking

Past research has often tried to foster counter-stereotypic content in the *current* self-concept [74, 79]. Yet, the results were mixed, and effects were, for example, mainly found on implicit measures [79, also see 80, 81]. In contrast to the (implicit or explicit) present self-concept, future-oriented self-conceptions, so-called possible selves, are less bound by current social feedback and by a need for consistency in self-descriptions [82]. As a result, possible selves are more malleable and can serve as means of identity exploration [83]. For men, it could thus be easier to consider counter-stereotypic communal roles for themselves in the future than in the present.

Markus and Nurius [82] proposed that possible selves can differ in valence by reflecting what an individual would like to become (desired self), is afraid to become (feared self), or expects to become (expected self). Consequently, possible selves can function as incentives and provide a framework against which behaviors and outcomes are evaluated [82]. Moreover, possible selves have been described as social products and are embedded in social identities [84, 85]. They not only provide a framework for personal identities but take into account category membership and what is possible for oneself as a group member [82, 84]. Hence, prototypes, representing the essence of a group, can be an important source for socially contextualized possible selves.

In addition to prototypes, the content of possible selves is likely also affected by salient life tasks and periods of transition such as parenthood [84, 86, 87]. Next to job-related possible selves, parenting constitutes an essential part of young parents’ conceptions of themselves in the future [87]. Also for men, parenting roles become increasingly important when transitioning to fatherhood. Yet, actual involvement in childcare also depends on the extent to which actual and possible selves overlap [88]. Even before becoming parents, women and men hope for rather role-congruent and fear rather role-incongruent possible selves in their distant future based on traditional social roles associating women more with caretaking and men more with breadwinning [89, also see 90]. However, research has shown that changing such gendered norms can facilitate the consideration of role-incongruent possible selves. In a previous study, priming women with male exemplars and men’s changing gender roles affected their possible selves: When women perceived men to increasingly participate in childcare, they expected more to engage in breadwinning (and vice versa) [91]. Yet, we are unaware of a study that tested the effect of changing masculinity norms on *men’s* possible selves and counter-stereotypic outcomes such as parental leave-taking.

## The present research

The aim of the present research was to examine different prototypical representations of men, so-called prototypes, as indicators of what it means to be a man, and their effect on men’s expectations and intentions regarding engagement in caretaking in the future. To that end, we focused on men’s possible selves and parental leave-taking expectations as communal outcomes in two experiments. We manipulated whether prototypical descriptions of men were characterized exclusively by agency, exclusively by communion, or by a combination of both to understand which compositions of agentic and communal content are most likely to increase men’s communal intentions. To test our predictions, we presented fictious newspaper articles about the ideal man of today (control condition: student or millennial of today, Exp. 1 vs. 2, respectively) to male students (Experiment 1) and to a broader male sample of participants (Experiment 2) who did not have children yet but planned to become parents in the future. We assessed possible selves via their possible self-concept (i.e., to what extent participants expected agentic and communal attributes to describe them in the future) and their possible task engagement (i.e., to what extent participants expected to engage in agentic and communal tasks and behaviors in the future) followed by an assessment of parental leave-taking intentions and additional variables.

In addition to these main goals of the research, we aimed to learn more about the mechanisms that affect men’s communal outcomes depending on different agentic and communal representations of their gender group. More specifically, we examined assimilation and contrast effects and affirmation and threat responses. However, we did not find substantial support for these assumptions (for results and discussion see S2 Text and General Discussion). To make these initial goals transparent, in Table 1 we present the original hypotheses described in the preregistrations (Exp. 1: https://aspredicted.org/tv34k.pdf; Exp. 2: https://aspredicted.org/2f69s.pdf) juxtaposed with the hypotheses described in the manuscript. Moreover, we report which analyses we had planned to conduct and where they are reported (in the manuscript or in S2 Text).

**Table 1:** Juxtaposition of hypotheses and analyses as presented in the preregistration in comparison to the manuscript for Experiments 1 and 2 (R = rephrased, B = broadened).

	Preregistration		Manuscript
**Experiment 1**			
*Hypotheses*			
**1**	Inclusive^1^ male prototypes should lead to more communal possible selves^2^ than in in the control condition (assimilation effect).	**1**	Presenting men with prototypes combining agency and communion leads to more communal outcomes compared to a control condition. *(r*, *b)*
**2**	Exclusively agentic male prototypes should lead to more communal possible selves than in the control condition.	**2**	Exclusively agentic prototypes of men should lead to more communal outcomes as compared to the control condition. *(b)*
**3**	Exclusively communal male prototypes should lead to more agentic possible selves than in the control condition.	**3.1**	Exclusively communal prototypes of men [should] lead to more agentic outcomes than in the control condition. *(b)*
**4**	Inclusive male prototypes should lead to more communal possible selves than exclusively communal male prototypes.	**4**	In any case, we expected prototypes of men combining agency and communion to lead to more communal outcomes than the exclusively communal prototype. *(r*, *b)*
**4**	However, the exclusively communal male prototype could also lead to assimilation effects if it is rather perceived as moderate than extreme.	**3.2**	However, given increasing integration of care into masculinity and fathering norms [32, 40, 42], prototypical representations of men focusing on communal attributes could not be perceived as extreme and thus rather be pulling men towards communal outcomes instead of pushing them away. *(r)*
*Analyses*			
	*Main analyses*: ANOVAs with planned contrasts as described in hypotheses		Reported in manuscript
	*Secondary analyses*: Mediation analyses examining whether possible selves mediate the relation between prototypes of men and parental leave-taking intentions		Reported in S2 Text
**Experiment 2**			
*Hypotheses*			
**1**	Male prototypes combining agency and communion lead to more communal outcomes than in the control condition.	**1**	Describing prototypical men as agentic and communal [should] increase men’s self-reported communal intentions as compared to the control condition. *(r)*
**2.1**	Male prototypes combining agency and communion lead to more communal outcomes than exclusively communal male prototypes.	**4**	We expected the combined agentic and communal prototype of men to lead to more communal outcomes than the exclusively communal prototype. *(r)*
**2.2**	Agentic male prototypes lead to more communal outcomes than the control condition.	**2**	For the prototypical representation of men focusing exclusively on agency, we again expected contrast effects in the form of more communal outcomes than in the control condition. *(r)*
**2.3**	Communal male prototypes do not lead to more communal outcomes than the control condition.	**3**	We did not expect any differences between the communal condition and the control condition on the dependent variables. *(r)*
**2.4**	We expect men to be more affirmed in their masculinity in the combined agentic and communal condition compared to the communal condition (and thus allowing for more communal outcomes). We expect men to be more threatened in the communal condition compared to the control condition and compared to the combined agentic and communal condition.		Hypothesis not included in manuscript (but see S2 Text)
**2.5**	We expect the combined agentic and communal prototype to be perceived as more moderate, ambiguous, and diverse than the exclusively agentic or communal prototypes and thus lead to assimilation (see 1.). On the contrary, we expect the exclusively agentic and the exclusively communal prototype to be perceived as more extreme, unambiguous, and one-sided than the combined agentic and communal prototype and thus lead to contrast (see 2.2., 2.3)^3^.		Hypothesis not included in manuscript (but see S2 Text)
*Analyses*			
	*Main analyses*: ANOVAs and planned contrasts as described in hypotheses		Reported in manuscript for hypotheses 1 to 2.3 and in S2 Text for hypotheses 2.4 and 2.5
	*Secondary analyses*: Mediation analyses to examine whether possible selves, threat, and affirmation mediate the relation between male prototypes and paternal leave-taking outcomes		Reported in S2 Text
	Moderation analyses to examine whether self-typicality and perceived extremity, ambiguity, and diversity moderate the relation between male prototypes and communal outcomes		Reported in S2 Text

^1^: In the preregistration of Experiment 1, we had used the more ambiguous term inclusive prototypes to describe prototypes combining agentic and communal content in contrast to prototypes exclusively containing agentic *or* communal content.

^2^: As we expected communal possible selves to mediate the relation between prototypes of men and men’s parental leave-taking outcomes, we only specified hypotheses for effects of prototypes of men on the mediator (possible selves) and failed to preregister hypotheses for direct effects on men’s parental leave-taking intentions and expected length of leave.

^3^: In hindsight, the hypothesis that the communal prototype of men should be perceived in line with and lead to contrast effects contradicts H2.3 in the preregistration and H3 in the manuscript which is why we dropped it.

## Experiment 1

In Experiment 1, we first tested the central hypothesis that presenting men with prototypes combining agency and communion leads to more communal outcomes compared to a control condition (H1). For all hypotheses, we define as more communal outcomes more communal possible selves (i.e., possible self-concept and possible task engagement), higher parental leave-taking intentions, and longer expected length of parental leave.

As prototypical representations of men focusing on only one dimension (agency *or* communion) could be interpreted as rather extreme, unambiguous, and one-sided, we expect contrast effects for these conditions. Accordingly, the second hypothesis was that exclusively agentic prototypes of men should lead to more communal outcomes as compared to the control condition (H2). For the communal condition, we had two possible hypotheses. In line with contrast effects and H2, exclusively communal prototypes of men could lead to more agentic outcomes than in the control condition (H3.1). However, given increasing integration of care into masculinity and fathering norms [32, 40, 42], prototypical representations of men focusing on communal attributes could not be perceived as extreme and thus rather be pulling men towards communal outcomes instead of pushing them away (H3.2). In any case, we expected prototypes of men combining agency and communion to lead to more communal outcomes than the exclusively communal prototype (H4).

### Method

The research plan was approved by the Ethics Commission of the Faculty of Psychology of the University of Koblenz-Landau (approval number 2019_200). We obtained informed consent by informing participants that by clicking the “next” button they agree to the study details as described in the consent form. We report how we determined sample size, all data exclusions, details on all conditions and all measures in the manuscript or in S4 Text.

#### Participants

The final sample size amounted to *N* = 132 participants and was thus sufficient to detect large-sized effects of *f* = 0.40 (η^2^ = 0.14) in a one-way ANOVA with four conditions according to the a-priori power analysis (based on previous results for the central contrast [combined agentic and communal vs. control condition]) [74]. This power analysis was conducted with G*Power 3 [92], an α of .05 and a statistical power of 1 - β = .95, which resulted in a necessary sample size of *N* = 112.

In total, we reached 334 men but screened out cases with more than 25% of missing values. Of the resulting 163 participants, we excluded several in advance based on our preregistered criteria. At the beginning of the survey, we screened out 10 participants because they did not self-identify as heterosexual and could therefore be subject to different norms. We administered additional preregistered exclusion criteria after data collection: We excluded the data of nine participants who failed an attention check (i.e., choosing “2” to show that they are reading carefully, interspersed in a measure of gender identification) and one person because he withdrew his approval for using his data for scientific purposes. No further participants were excluded based on the criteria that they already had children, did not want children in the future, were not students, or failed quality or suspicion checks. Outlier analyses based on Cook’s distance led to the exclusion of 11 cases (see S3 Text for results including outliers).

The final sample had an average age of 26 years (*M* = 25.53, *SD* = 4.52), ranging between 19 and 47 years. Regarding their highest level of education, 55% had graduated from high school, 38% had a university degree, and 6% had completed an apprenticeship. Most participants indicated being single (48%) or in a committed relationship (48%). Fields of study included psychology (13%), educational sciences and teaching (9%), computer science (9%), business (8%), and sciences (8%).

#### Procedure and experimental manipulation

We recruited participants online via university mailing lists, social media, and a commercial panel. They were invited to take part in a study on the evaluation of newspaper articles and had the chance to win 200€ in total in a lottery. After agreeing to the informed consent, the exclusion criteria specified above were queried. Then, we assigned the remaining participants randomly to one of the four conditions presenting different prototypical representations of men: combined agentic and communal prototype (*n* = 30) vs. agentic prototype (*n* = 35) vs. communal prototype (*n* = 37) vs. control condition (*n* = 30). In each condition, the participants read a contrived newspaper article (for full materials, see https://osf.io/ah9v4/). In all conditions, the article was framed by describing current debates and insecurities regarding what constitutes the ideal man of today (control condition: ideal student of today) as well as the results of an investigation to gain insight into this question. In the experimental conditions, masculinity was–according to the results of the journalists’ research–defined nowadays by varying degrees of agency and communion depending on the condition and affirmed accordingly. The agentic and communal attributes mainly indicated the presence of the respective content, not their absence (i.e., “assertive” indicating the presence of agency instead of attributes indicating its absence such as “aimless”) [93]. Moreover, we included attributes from different subdimensions of agency (assertiveness and competence) and communion (warmth and morality) [94]. For the combined agentic and communal condition, we included two versions with reversed order of agentic and communal attributes. In the communal condition, we included a few negative attributes (i.e., gullible, subordinates self) [70, 95] to counterbalance the more positive rating of this condition compared to the other conditions as indicated by a pre-test. The control condition included the same parts as the experimental conditions. However, as our sample consisted only of students, the student of today, as an ingroup prototype, was described in the control condition, and the description included as neutral content as possible. This implies that we did not use any gendered pronouns (however, we also refrained from using the gender-sensitive form of the German word “Student”, instead using what is called the generic masculine form). As agency and communion are so universal, some of the content could be matched to these fundamental content dimensions (e.g., students spending time on their hobbies or working as student assistants). Nevertheless, the experimental conditions all mentioned aspects of work and family or social life but with regard to agentic or communal content (i.e., men being caretakers in their family in the communal condition vs. being breadwinners in the agentic condition). In the control condition, this relation to agentic or communal content was omitted. Following the manipulation, the participants completed the dependent variables, manipulation checks, and further variables that are not the subject of the present research (desired and feared possible selves, current self-concept, desired (length of) parental leave-taking, perceived self-efficacy for parental leave-taking, feeling comfortable communicating parental leave-taking plans, perceived compatibility of agency and communion, distinctiveness threat, perceived diversity of men, gender identification, gender role attitudes, perceived pressure to fulfill agentic and communal roles; see S4 Text for details). At the end of the survey, we assessed demographic information, informed the participants about the design and purpose of the study, and gave them the chance to withdraw their approval for using their data for scientific purposes.

#### Measures

After the manipulation, we first checked the general *perception of the prototypes* presented in the newspaper articles. Specifically, we asked participants to indicate their spontaneous impression of the given description of men (students) as negative versus positive on a scale from 0 to 100.

Next, we assessed the dependent variables pertaining to possible selves. First we assessed the *possible self-concept* via close-ended measures following Oyserman and Markus [96]; yet, using agentic and communal traits. Participants rated the extent to which five agentic (e.g., independent, competitive; α = .61) and five communal traits (e.g., emotional, understanding; α = .74; based on the GEPAQ) [69, 95] were likely to describe them in 15 years or around the time when they want to have children on a 5-point Likert scale ranging from 1 (*not at all*) to 5 (*very much*).

To include a more task- and behavior-oriented operationalization, we measured *possible task engagement* via three work-related behaviors (e.g., going to work; not aggregated to form a scale as α = .41) and three family-related behaviors (e.g., taking care of children; α = .73; based on the Gender Role Behavior Scale and previously used tasks to assess possible selves) [91, 97]. Participants indicated on a 5-point scale to what extent they expect that these behaviors will be typical for them in 15 years or when they expect to have children.

We then measured outcome variables related to parental leave. To ensure that all participants had the same background knowledge, the participants first read a short text on parental leave policies in Germany. Afterwards they imagined that they would have children and had to decide on whether to take parental leave. The participants indicated their *parental leave-taking intentions* on a 7-point scale ranging from 1 (*not at all*) to 7 (*very much*) (“I plan to take parental leave”) [adapted from 98]. In addition, they indicated *how long they expected to take parental leave* (0 to 12 months).

As the *manipulation check*, we lastly assessed to what extent four agentic (e.g., assertive, competent; α = .72) and four communal (e.g., caring, trustworthy; α = .71) attributes described the man of today (control condition: student of today) according to the article they read in the beginning [93].

### Results

After checking assumptions and screening the data, we conducted one-way ANOVAs to examine the main effect of condition followed up by planned contrasts. In cases of variance heterogeneity, we conducted Welch tests and pairwise *t*-tests as post-hoc analyses. Because we only included hypotheses for agentic outcomes for the communal prototype of men (vs. the control condition; H3.1), we conducted *t*-tests for this comparison. For ANOVAs and *t*-tests, we used the Benjamini-Hochberg method [99] to control the false discovery rate and report cases in which *p*-values equal or exceed .05 after corrections. As effect sizes, we report eta-squared for ANOVAs and Cohen’s *d* for planned contrasts including 90% and 95% confidence intervals respectively. When *p*-values fall above .05 and the confidence intervals of effect sizes include zero, we interpret the results as non-significant. All analyses were conducted with and without participants who failed attention, suspicion, or quality checks, and with and without cases with a Cook’s distance larger than 4/*n*. We report results in the manuscript with these cases excluded and report when results differ with inclusions in S3 Text.

#### Manipulation check

The manipulation check showed that the manipulation was perceived as intended (see Table 2 for descriptive statistics). First, participants perceived different degrees of *agency* in the presented prototypes of men, *F*(3, 128) = 19.27, *p* < .001, η^2^ = .31, [.20; .40]. As planned, the agentic condition, *p* < .001, *d* = 0.87, [0.51; 1.24], and the combined agentic and communal condition, *p* < .001, *d* = 0.62, [0.27; 0.98], were perceived as more agentic than the control condition. The communal condition was perceived similarly as the control condition on agency, *p* = .149, *d* = -0.26, [-0.60; 0.09].

**Table 2:** Means and standard deviations for manipulation check and perception of prototypes in experimental conditions (Experiment 1).

	Experimental Condition			
	*Control*	*Communion*	*Agency*	*Agency & Communion*
Agency^1^	4.80 (0.99)	4.42 (1.14)	6.11 (1.00)	5.78 (1.12)
Communion^1^	4.78 (0.77)	5.95 (0.96)	3.66 (1.05)	5.79 (1.15)
Negative–positive^2^	65.13 (24.54)	69.05 (22.86)	44.09 (27.50)	63.97 (27.87)

Means with standard deviations in parentheses.

^1^: Scale from 1 to 7

^2^: Scale from 0 to 100.

Second, participants also perceived different degrees of *communion* in the presented prototypes of men, *F*(3, 128) = 39.16, *p* < .001, η^2^ = .48, [.37; .56]. As planned, the communal condition, *p* < .001, *d* = 0.84, [0.48; 1.20], and the combined agentic and communal condition, *p* < .001, *d* = 0.69, [0.34; 1.05], were perceived as more communal than the control condition. The agentic condition was perceived as less communal than the control condition, *p* < .001, *d* = -0.80, [-1.16; -0.44].

In addition, we examined how negative versus positive the experimental conditions were perceived and indeed found substantial differences, *F*(3, 128) = 6.63, *p* < .001, η^2^ = .13, [.04; .22]. According to the participants, the description of the man of today in the agentic condition was more negative than in the combined agentic and communal condition, *p* = .009, *d* = -0.72, [-1.22; -0.22], than in the communal condition, *p* < .001, *d* = -0.99, [-1.48; -0.50], and than in the control condition, *p* = .006, *d* = -0.80, [-1.31; -0.30]. The other conditions were not perceived substantially differently from each other, all *p*s > .633. We also found substantial differences as to how pleasant, extreme, and desirable the prototypical representations of men were perceived (see S2 Text for results).

#### Dependent variables

Descriptive statistics for all dependent variables can be found in Table 3. In general, the sample showed high communal expectations: Across all conditions, possible selves measures ranked around 4 on a 5-point scale and average parental leave-taking intentions varied between 4.77 and 5.93 on a 7-point scale. Also, the expected length of parental leave was consistently above the average leave-taking period of fathers in Germany of roughly three months [100] (which had not been made explicit to participants). In the combined agentic and communal condition, participants even expected to take nearly eight months of parental leave. As parents can divide 14 months between themselves if each partner takes at least two months, more than seven months would represent a longer leave for fathers than mothers.

**Table 3:** Means and standard deviations for possible selves and parental leave outcomes in experimental conditions (Experiment 1).

	Experimental Condition			
	*Control*	*Communion*	*Agency*	*Agency & Communion*
Communal possible self-concept^1^	3.75 (0.60)	3.76 (0.71)	3.80 (0.47)	3.97 (0.63)
Agentic possible self-concept^1^	3.67 (0.54)	3.67 (0.67)	3.78 (0.70)	3.67 (0.53)
Communal PTE^1^	3.74 (0.68)	3.96 (0.70)	4.13 (0.62)	4.26 (0.67)
Agentic PTE (going to work) ^1^	4.37 (0.76)	4.27 (0.80)	4.49 (0.70)	4.17 (0.91)
Agentic PTE (other household tasks) ^1^	3.87 (0.94)	3.65 (1.16)	3.46 (1.09)	4.07 (0.87)
Agentic PTE (working overtime) ^1^	3.27 (1.17)	3.11 (1.02)	3.20 (1.02)	3.37 (1.00)
Parental leave-taking intentions^2^	4.77 (1.77)	4.84 (1.72)	5.71 (1.58)	5.93 (1.14)
Expected length of parental leave^3^	5.20 (3.67)	5.83 (3.55)	6.09 (3.76)	7.77 (3.13)

PTE = Possible task engagement. Means with standard deviations in parentheses.

^1^: Scale from 1 to 5

^2^: Scale from 1 to 7

^3^: Scale from 0 to 12 (months).

Regarding hypothesis tests, we found general support for the hypotheses for parental leave-taking variables but less so for possible selves. For the first operationalization of possible selves, the *possible self-concept*, we did not find substantial differences between the experimental conditions. Specifically, whether the presented prototypes of men were described as agentic, communal, or both did not substantially affect men’s self-reported communal possible self-concept, *F*(3, 128) = 0.88, *p* = .455, η^2^ = .02, [.00; .06], or agentic possible self-concept, *F*(3, 128) = 0.29, *p* = .830, η^2^ < .01, [.00; .02].

However, how prototypes of men were described affected the second operationalization of possible selves, *possible task engagement*, *F*(3, 128) = 3.41, *p* = .020, η^2^ = .07, [.01; .14]; yet the adjusted empirical *p*-value was .050. When the man of today was described via a combination of agency and communion, men expected communal tasks to be more typical for themselves in the future than in the control condition, *p* = .004, *d* = 0.52, [0.17; 0.88], but not substantially more than in the communal condition, *p* = .069, *d* = 0.44, [-0.05; 0.92]. These findings support H1 that combined agentic and communal prototypes of men should lead to more communal self-reported intentions than in the control condition, but contradict H4 as the comparison to the communal condition was not significant. Regarding H2 that a contrast effect for the agentic condition should also increase communal intentions, men expected communal tasks to be more typical for themselves in the future in the agentic condition as compared to the control condition, *p* = .020, *d* = 0.41, [0.06; 0.76], but this comparison was not significant when outliers were included. Contrary to H3.1 and H3.2, presenting solely communal prototypes of men neither substantially affected men’s self-reported communal nor agentic possible selves regarding task engagement, *p*s > .495, *d*s < 0.23. These results held when controlling for age, educational level, and relationship status in a hierarchical regression. The model including the experimental conditions (dummy-coded with the control condition as the reference group) explained significantly more variance in the dependent variable than the base model, *F*(3, 119) = 5.04, *p* = .003, Δ*R*^2^ = .09.

**Fig 1:**
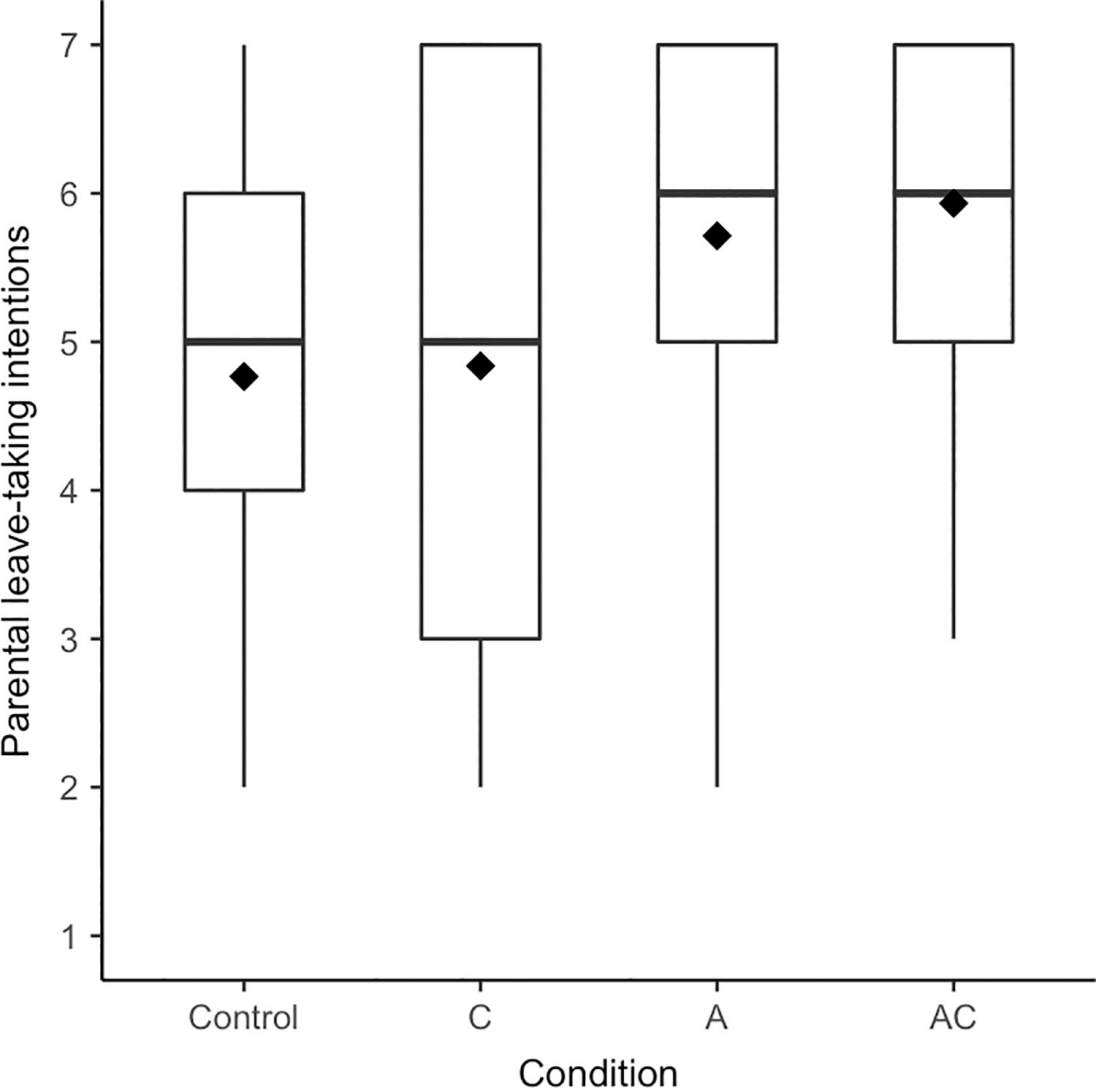
Boxplots for parental leave-taking intentions separated by condition (Experiment 1). Diamonds represent means, horizontal lines represent medians.

Boxplots for *parental leave-taking intentions* are presented in Fig 1. As expected, presenting different prototypes of men affected men’s self-reported parental leave-taking intentions, *F*(3, 128) = 4.58, *p* = .004, η^2^ = .10, [.02; .17]. In line with H1 and H4, when the man of today was described via a combination of agency and communion, men reported planning more to take parental leave than in the control condition, *p* = .005, *d* = 0.50, [0.15; 0.85], and than in the communal condition, *p* = .006, *d* = 0.73, [0.24; 1.23]. A solely agentic prototypical representation of men was linked to higher parental leave-taking intentions as compared to the control condition, *p* = .018, *d* = 0.42, [0.07; 0.77], supporting H2. When the man of today was only defined via communion, men’s parental leave-taking intentions did not substantially differ from those in the control condition, *p =* .855, *d* = 0.03, [-0.31; 0.38], thus not supporting H3.2. Again, the results held when including controls in a first step, and the second step’s model including the experimental conditions explained significantly more variance in parental leave-taking intentions, *F*(3, 119) = 3.74, *p* = .013, Δ*R*^2^ = .06.

**Fig 2:**
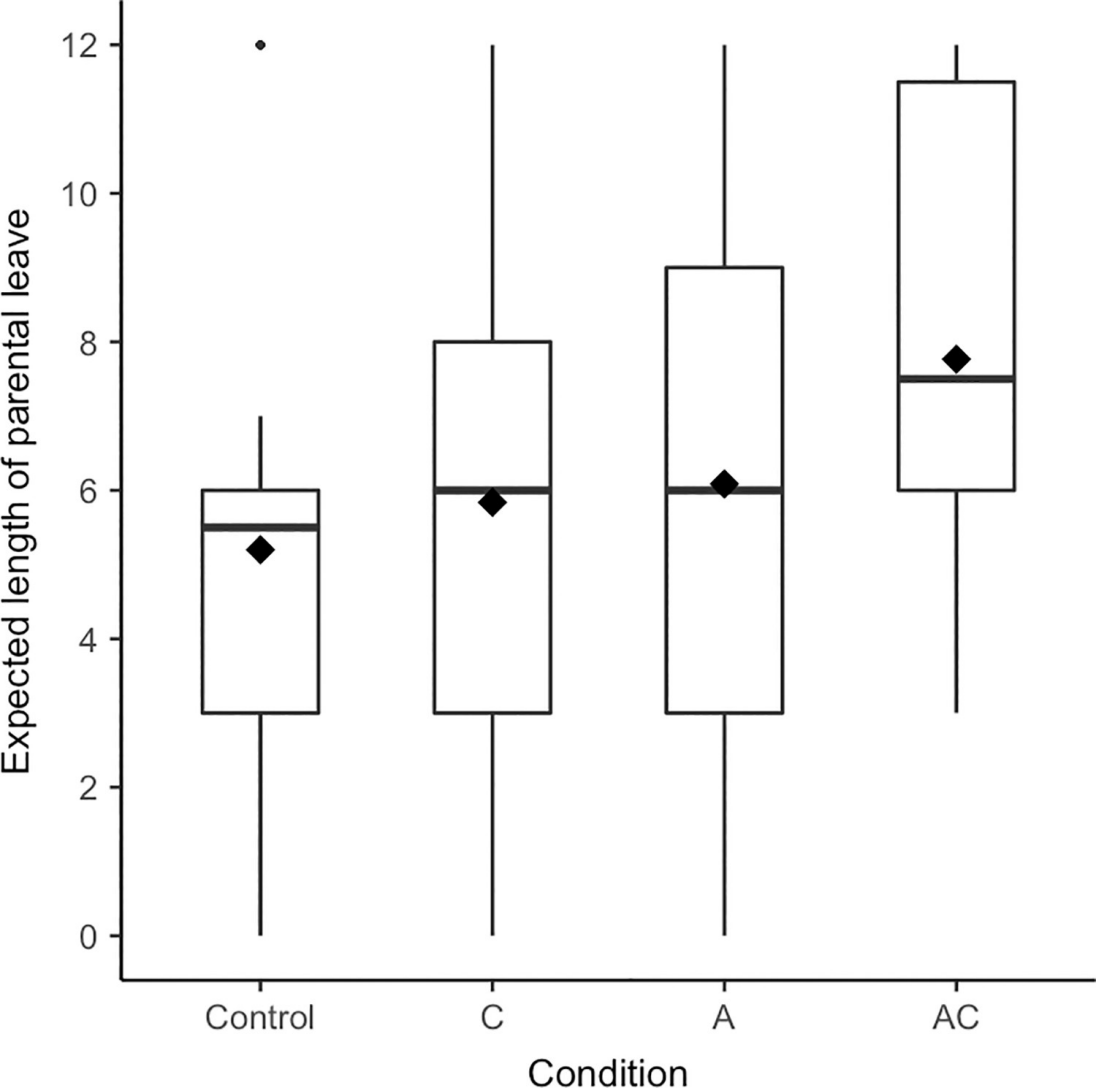
Boxplots for expected length of parental leave separated by condition (Experiment 1). Diamonds represent means, horizontal lines represent medians.

Men’s self-reported *expected length of parental leave* also tended to be affected by the experimental conditions, *F*(3, 128) = 2.89, *p* = .038, η^2^ = .06, [.00; .13] (see Fig 2); *p*_adjusted_ = .063. When the man of today was described via a combination of agency and communion, men reported to expect taking longer leave than in the control condition, *p* = .006, *d* = 0.50, [0.14; 0.85], and the communal condition, *p* = .029, *d* = 0.58, [0.09; 1.07]. Men’s expected length of parental leave did not substantially differ from the control condition when the man of today was solely defined via agency *or* via communion, *p*s > .317, *d*s < 0.18. The model including the experimental conditions descriptively explained more variance in the dependent variable than the base model but this difference was not statistically significant, *F*(3, 119) = 2.48, *p* = .064, Δ*R*^2^ = .04. Moreover, the confidence interval of the omnibus *F*-test’s effect size included 0 and the adjusted *p*-value was .063. As we therefore cannot conclude that an effect is present, we tested for the absence of a meaningful effect by examining whether the observed effect is smaller than a smallest effect size of interest (SESOI) [101–103]. We determined the SESOI based on the study we used for the power analysis in Experiment 1 [74] following Simonsohn’s [104] approach [102]. Based on the results of the equivalence test, *p* = .850, we cannot reject the H0 that there are meaningful differences between the experimental conditions, meaning that the obtained effect size (η^2^ = .063) appears to be larger than the SESOI (η^2^ = .014). Thus, the differences are neither clearly statistically different nor statistically equivalent and thereby inconclusive, likely due to a lack of power [102, 103]. In sum, presenting combined agentic and communal prototypes of men only tended to lead to a longer expected length of parental leave compared to the control condition, providing tentative support for H1. In addition, we only found tentative support for H4 and no support for H2 and H3.2.

### Discussion

The aim of Experiment 1 was to gain insight into which kinds of prototypical representations of men can draw men towards communal outcomes such as more communal self-reported possible selves and parental leave-taking intentions. Specifically, we tested the central hypothesis (H1) that presenting a description of the man of today as agentic *and* communal would be effective in fostering communal intentions in men. We found (partial) support for this hypothesis on all dependent variables except for the possible self-concept. When the man of today was described as agentic and communal, men expected communal tasks to be more typical for themselves in the future (possible task engagement), had higher parental leave-taking intentions, and tended to expect taking longer parental leave than in the control condition. We also found partial support for the supplementary Hypothesis 2: In line with contrast effects, describing the man of today as solely agentic led to men’s higher self-reported parental leave-taking intentions and more communal possible task engagement (when outliers were excluded). Presenting exclusively communal prototypes of men neither affected men’s agentic (H3.1) nor communal outcomes (H3.2). Lastly, presenting a combination of agency and communion in prototypes of men resulted in higher self-reported parental leave-taking intentions (when outliers were excluded) and longer expected leave not only as compared to the control condition but also as compared to the communal condition (H4).

As we found initial support for the main hypothesis on all other dependent variables, the question remains why prototypical representations of men did not affect the possible self-concept. It is possible that being understanding and warm to others in the future is a positive outlook that participants are motivated to claim for themselves (regardless of experimental conditions). This assumption is in line with research on self-enhancement that shows that people have generally optimistic views about themselves and their futures [105, 106]. Moreover, we asked participants to rate their possible self-concepts around the time when they want to have children. Parenthood implies caretaking which makes it likely that participants expect themselves to be communal in the future, when they are parents. In other words, our measure for the possible self-concept may not have allowed for much variance and was thus adapted in Experiment 2. We did find differences for possible task engagement between the experimental conditions for which we chose a more relational approach by asking what behaviors participants expect to be more typical for themselves in the future. These differences in the assessment of both operationalizations of possible selves may explain our different findings.

Besides the main hypothesis, the expected contrast effects were mainly found for the exclusively agentic and not the exclusively communal prototype of men. Based on the assessed perceptions of the prototypes (also see S2 Text), the prototypical description of men focusing on communal attributes was not perceived very differently than the control condition and was perceived much more positively than the prototypical description of men focusing on agentic attributes (even though we tried to counteract this positivity bias). These perceptions and the generally high communal intentions suggest that for our sample, men’s communal engagement was not perceived as highly non-normative. A possible explanation is the student sample (including many education and psychology majors) who could differ from the general population in their attitudes and actual experience with leave-taking considerations [107, 108]. Further limitations of Experiment 1 can be found in the measurement of the central variables. For example, the possible task engagement only included few tasks which did not always form reliable scales. The ceiling effects we obtained for possible task engagement could be related to the phrasing of the items: Asking how typical tasks will be in the future, leaves room for interpretation regarding what “typical” means, and men could overestimate their engagement (as compared to other men, rather than women, i.e., shifting standards) [109, 110] with ceiling effects as a result. We addressed these issues in a second experiment.

## Experiment 2

The goal of Experiment 2 was to reexamine the predictions in a larger and more diverse sample and improve several of the measures used. Recruiting from the general population and not only students meant that we had to adapt the control condition, previously describing the student of today. To address another ingroup prototype, we created a new control condition on the millennial of today (for full materials, see https://osf.io/ah9v4/). We again aimed to recruit men who did not yet have children but wanted to become parents in the future, and especially targeted employees for whom taking parental leave may be more relevant soon. Thus, we targeted participants who would be part of the generation of millennials (being born in the 1980s or 1990s).

We changed several aspects of the possible selves measures. To reduce ceiling effects and allow for more variance, we used 7-point scales for all possible selves measures, which previously used 5-point scales. Moreover, based on recent insights into gender stereotypes [39], we only used sub-dimensions of communion for the possible self-concept on which in previous research women and men differed in self-ratings to further prevent ceiling effects. For the possible task engagement, we adapted the instructions so that participants were asked to indicate *how often* they expected to engage in certain tasks in the future. We assumed that asking about frequencies rather than typicality of tasks would represent a more objective response format [109, 110]. In addition, we did not ask participants to imagine their future 15 years from now but more generally around the time when they wanted to have children (as this could be sooner for many). Before the parental leave variables, we again included a text on current policies in Germany but made some adaptations to not communicate current norms and potentially push participants into a certain direction. Furthermore, we added three items to our previously single item measure of parental leave-taking intentions to ensure a more valid and sensitive assessment.

The main hypothesis was again that describing prototypical men as agentic and communal would increase men’s self-reported communal intentions as compared to the control condition (H1). We defined communal outcomes in the preregistration as more communal possible selves, higher parental leave-taking intentions, and longer expected length of parental leave. For the prototypical representation of men focusing exclusively on agency, we again expected contrast effects in the form of more communal outcomes than in the control condition (H2). For the prototypical representation of men focusing exclusively on communion, the lack of substantial findings in Experiment 1 suggests, as expected, that an exclusive focus on communion could simultaneously draw men towards communal outcomes and push them away, with effects cancelling each other out. Thus, we did not expect any differences between the communal condition and the control condition on the dependent variables (H3) and only included the communal prototypical description of men to replicate the findings from Experiment 1. Moreover, we only examined communal outcomes (instead of both agentic and communal ones for this condition as in Experiment 1). Yet, as in Experiment 1, we expected the combined agentic and communal prototype of men to lead to more communal outcomes than the exclusively communal prototype (H4).

### Method

The research plan was again approved by the Ethics Commission of the Faculty of Psychology of the University of Koblenz-Landau (approval number 2019_200), and informed consent was obtained as in Experiment 1 within the online survey. We again report how we determined sample size, all data exclusions, details on all conditions and all measures in the manuscript or in S4 Text.

#### Participants

Based on the medium-sized effects found in Experiment 1, we conducted an a-priori power analysis for obtaining medium-sized effects of *f* = 0.25 (η^2^ = .06) in a one-way ANOVA. With α = .05 and a statistical power of 1 –β = .95, a sample size of *N* = 280 was required. We recruited 322 male participants of which we excluded upfront–as preregistered–three participants who were already parents and seven participants who did not want to have children. For sexual orientation, we softened our preregistered exclusion criteria to also include participants who mainly feel attracted to women or who feel attracted to women and men equally. Sixteen participants did not meet the sexual orientation criterion. Another 44 participants withdrew their approval for using their data for scientific purposes and were thus excluded. This relatively high number of withdrawals likely occurred because initially participants could not reverse clicking the item for withdrawing the approval to use their data (e.g., when clicking first and then reading the instruction). After participants informed us about this and we enabled unclicking the item, the number of subsequent withdrawals decreased. Lastly, we excluded three participants who failed an attention check (interspersed in the measure of parental leave-taking intentions), one participant who failed a quality check, and 15 outliers based on Cook’s distances larger than 4/*n* (results including outliers are again presented in S3 Text). The final sample size comprised *N* = 233 participants which corresponds to a statistical power of .90 to detect medium-sized effects given our design.

The final sample was on average 26 years old (*M* = 25.55, *SD* = 4.87) with a range from 18 to 48 years. Most participants were highly educated: 42% held a university degree, 40% had graduated from high school, and 11% had completed an apprenticeship. Fifty-one percent of participants were students, 39% were employees, and 4% were apprentices or pupils. Regarding their relationship status, 52% indicated being in a committed relationship, 42% were single, and 4% married or in a registered civil partnership.

#### Procedure and experimental manipulation

The procedure was similar to Experiment 1 with the following exceptions. The recruitment took place via mailing lists, social media, face-to-face recruitment in a city center of a small town in South-West Germany, and through personal contacts of student assistants. We aimed for a more diverse sample beyond students and especially targeted employed participants who were more likely to take parental leave soon.

We invited participants to a study regarding plans for their future and raffled 120€ in prizes in total. After informed consent and exclusion criteria, the participants were again randomly assigned to one of the four conditions including different prototypical representations of men varying in agentic and communal content (combined agentic and communal, *n* = 62, vs. agentic, *n* = 54, vs. communal, *n* = 56, vs. control condition, *n* = 61). As we were aiming for a more diverse sample, we adapted the control condition, previously describing a student prototype, to represent an ingroup prototype for the majority of participants: the millennial of today. As for the control condition in Experiment 1, we mostly refrained from using gendered pronouns by using plural forms and again mentioned aspects of the work and family or social life but without complementing them with agentic or communal content. Again, the article claimed to describe an ideal image of the millennial of today (yet the term “ideal” was mentioned once instead of twice as in the other conditions).

Moreover, we refrained from adjusting the positivity bias in the communal condition. Lastly, we did not include two versions of the combined agentic and communal condition with reversed order of agentic and communal attributes anymore for the sake of simplicity but mixed the order throughout the manipulation.

After the manipulation, the participants again completed the dependent variables, manipulation checks, and further variables (closeness between the self, men, and millennials, current self-concept, agentic possible self-concept and possible task engagement, second operationalization of possible selves, affirmation of masculine identity, prototypicality threat, threat-related emotions, self-typicality, perceived care-giving competence, ambivalent sexism; see S4 Text for details). At the end of the survey, we assessed demographic information, debriefed participants, and offered a chance to withdraw their approval for using their data for scientific purposes.

#### Measures

Unless otherwise indicated, we used 7-point scales ranging from 1 (*strongly disagree*) to 7 (*strongly agree*) in a German-language survey. After the manipulation, we again checked the *perception of the presented prototypes* as negative versus positive on a scale from 1 to 10.

Next, we assessed the *possible self-concept* again via close-ended measures following Oyserman and Markus [96]. We included seven communal attributes for the subdimensions concern for others (e.g., compassionate) and emotional sensitivity (e.g., emotional) respectively, which we combined to form an overall scale (α = .79) [39].

For *possible task engagement*, we asked participants additionally to rate how often they expected to engage in gender role relevant tasks around the time when they wanted to have children (7-point scale ranging from 1 = *never* to 7 = *very often*). We focused the analyses on communal tasks which we especially defined as childcare tasks (e.g., physical care of child; α = .67). Routine housework tasks were analyzed secondarily (e.g., preparing food; α = .59) [adapted from 91, 111–113].

After participants read a short information text on parental leave policies in Germany, we assessed *parental leave-taking intentions* now with four items (e.g., “I will probably take parental leave.”; α = .93) [adapted from 98, 114]. *Expected length of parental leave* was assessed as in Experiment 1 (open-answering format with possible answers between 0 and 12 months). The *manipulation check* was also identical to Experiment 1 (α_agency_ = .84, α_communion_ = .86).

### Results

We followed the same analysis strategy as in Experiment 1.

#### Manipulation check

As in Experiment 1, the manipulation was perceived as intended (see Table 4 for descriptive statistics). First, participants perceived different degrees of *agency* in the presented prototypes of men, *F*(3, 229) = 18.80, *p* < .001, η^2^ = .20, [.12; .27]. The prototype in the agentic condition, *p* < .001, *d* = 1.19, [0.79; 1.59], and in the combined agentic and communal condition, *p* = .003, *d* = 0.58, [0.22; 0.94], was perceived as more agentic than in the control condition. The prototype of men in the communal condition, *p* = .567, *d* = 0.12, [-0.24; 0.48], was perceived similarly as in the control condition on agency.

**Table 4:** Means and standard deviations for manipulation check and perception of prototypes in experimental conditions (Experiment 2).

	Experimental Condition			
	*Control*	*Communion*	*Agency*	*Agency & Communion*
Agency^1^	4.53 (1.04)	4.65 (0.97)	5.91 (1.28)	5.13 (1.03)
Communion^1^	4.44 (0.95)	5.96 (0.85)	3.89 (1.33)	5.53 (0.99)
Negative–positive^2^	6.62 (1.82)	7.89 (1.55)	5.54 (2.54)	7.71 (1.69)

Means with standard deviations in parentheses.

^1^: Scale from 1 to 7

^2^: Scale from 1 to 10.

Second, the presented prototypes of men also differed by condition regarding *communion* according to the participants, Welch’s *F*(3, 124.76) = 47.07, *p* < .001, η^2^ = .53, [.43; .61]. The prototype in the communal condition, *p* < .001, *d* = 1.68, [1.26; 2.10], and in the combined agentic and communal condition, *p* < .001, *d* = 1.12, [0.74; 1.50], was perceived as more communal than in the control condition. The prototype of men in the agentic condition was perceived as lower on communion than in the control condition, *p* = .014, *d* = -0.48, [-0.85; -0.11].

We again examined how negatively versus positively the presented prototypes were perceived and found substantial differences, *F*(3, 108.32) = 13.12, *p* < .001, η^2^ = .27, [.14; .37]. Participants perceived the description of the man of today in the agentic condition as more negative than in the combined agentic and communal condition, *p* < .001, *d* = -1.03, [-1.44; -0.62], than in the communal condition, *p* < .001, *d* = -1.14, [-1.56; -0.71], and than in the control condition, *p* = .008, *d* = -0.49, [-0.90; -0.09]. They further perceived the description of the man of today in the combined agentic and communal condition as more positive than in the control condition, *p* = .005, *d* = 0.62, [0.24; 1.01], but not substantially differently from the communal condition, *p* = .621, *d* = -0.11, [-0.48; 0.26]. Lastly, the description in the communal condition was perceived as more positive than in the control condition, *p* = .002, *d* = 0.75, [0.35; 1.15]. We also found substantial differences as to how extreme, unambiguous, and one-sided the prototypical representations of men were perceived (see S2 Text for results).

#### Dependent variables

Descriptive statistics for all possible selves and parental leave-taking variables can be found in Table 5. Although we tried to prevent ceiling effects, participants expected to be highly engaged in caretaking and parental leave in the future in all conditions (all mean ratings between 5 and 6 on a 7-point scale). Regarding the expected length of parental leave, ratings fluctuated around six months. Thus, on average, participants indicated expecting an almost equal division of parental leave-taking between their partners and themselves (given that in Germany, 14 months of paid leave are available to share between partners if each takes at least two months).

**Table 5:** Means and standard deviations for possible selves and parental leave outcomes in experimental conditions (Experiment 2).

	Experimental Condition			
	*Control*	*Communion*	*Agency*	*Agency & Communion*
Possible self-concept^1^	5.49 (0.84)	5.48 (0.79)	5.51 (0.84)	5.46 (0.86)
PTE–childcare^1^	5.60 (0.88)	5.70 (0.74)	5.84 (0.77)	5.68 (0.88)
PTE–housework^1^	5.11 (0.90)	5.20 (1.10)	5.22 (0.96)	5.26 (1.00)
Parental leave-taking intentions^1^	5.25 (1.41)	5.59 (1.34)	5.99 (1.01)	5.78 (1.28)
Expected length of parental leave^2^	5.87 (3.92)	5.96 (3.76)	6.59 (3.67)	6.23 (3.83)

PTE = Possible task engagement. Means with standard deviations in parentheses.

^1^: Scale from 1 to 7

^2^: Scale from 0 to 12 (months).

To anticipate, the results again tended to be stronger for parental leave-taking outcomes than possible selves. For possible selves, neither presenting the combined agentic and communal nor the exclusively agentic prototypes of men led to more self-reported communal *possible self-concepts*, *F*(3, 229) = 0.03, *p* = .995, η^2^ < .01, [.00; .00], or more communal *possible task engagement*, *F*s < 0.86, η^2^s < .02. Varying degrees of agency and communion in presented prototypes of men did not substantially affect how men saw themselves in the future or their expectations for engaging in different roles around the time when they wanted to have children. Thus, the results do not support the hypotheses that a combined agentic and communal prototypical representation of men (H1) or an agentic prototypical representation of men (H2) lead to more communal intentions (i.e., communal possible selves here) as compared to the control condition (or as compared to the communal condition for the combined agentic and communal prototype; H4). These findings are consistent with H3, that presenting communal prototypes of men would not lead to more communal possible self-concepts, *p* = .974, *d* = -0.01, [-0.38; 0.35], or possible task engagement, *p*s > .485, *d*s < 0.14, than the control condition.

**Fig 3:**
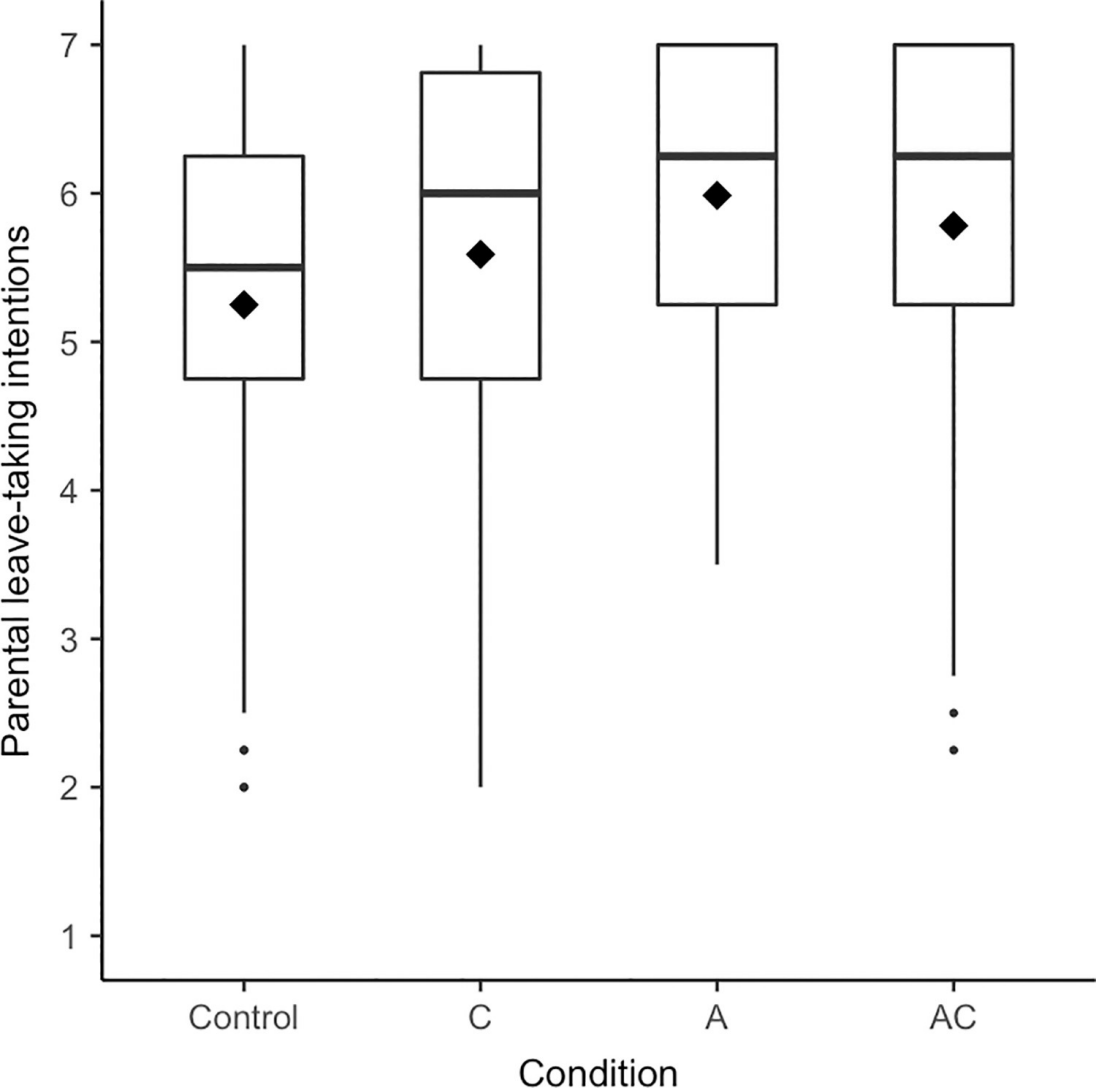
Boxplots for parental leave-taking intentions separated by condition (Experiment 2). Diamonds represent means, horizontal lines represent medians.

Regarding men’s parental leave-taking, somewhat more support for the hypotheses was found–especially for leave-taking intentions. Participants differed in their reported *parental leave-taking intentions* depending on experimental condition, *F*(3, 229) = 3.51, *p* = .016, η^2^ = .04, [.00; .09]; yet the adjusted *p*-value was .080 (see Fig 3 for boxplots). In line with the main hypothesis (H1), participants reported higher parental leave-taking intentions when the man of today was described as agentic and communal compared to the control condition, *p* = .022, *d* = 0.39, [0.04; 0.75]. Yet, contrary to H4, the comparison to the communal condition was not significant, *p* = .402, *d* = 0.15, [-0.22; 0.51]. We found support for contrast effects in the agentic condition (H2): Presenting exclusively agentic prototypes of men also led to higher parental leave-taking intentions than the control condition, *p* = .002, *d* = 0.60, [0.22; 0.97]. In line with H3, exclusively communal prototypes did not substantially affect men’s parental leave-taking intentions compared to the control condition, *p* = .152, *d* = 0.25, [-0.12; 0.61]. We again tested whether a model including the experimental conditions explained more variance in parental leave-taking intentions than a base model including age, employment status, and relationship status as controls. This was indeed the case, *F*(3, 211) = 3.27, *p* = .022, Δ*R*^2^ = .03. Given the adjusted *p*-value of the omnibus *F*-test and the effect size’s confidence interval including zero, we also conducted an equivalence test which revealed that we cannot rule out that meaningful effects are present, *p* = .837.

**Fig 4:**
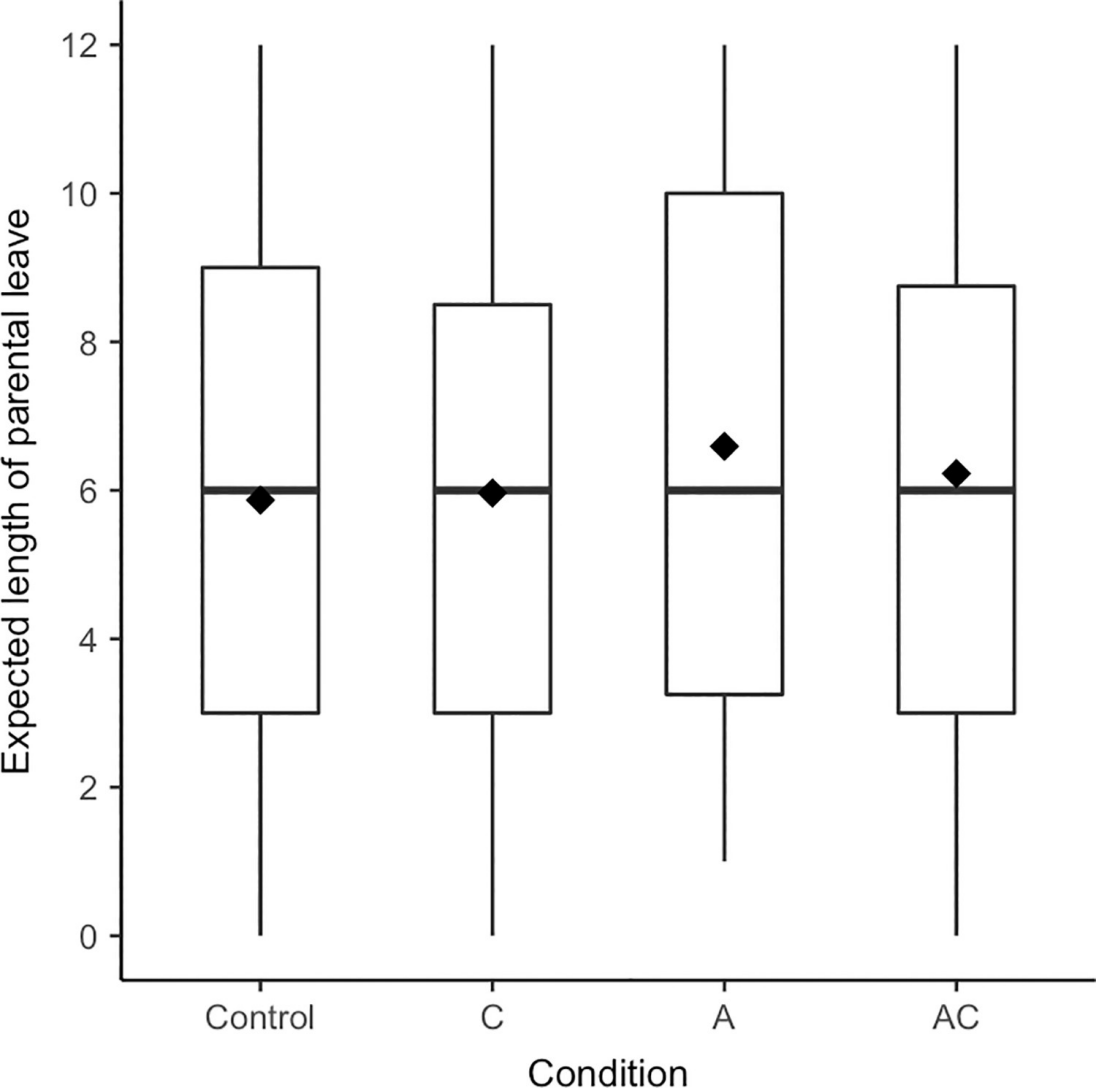
Boxplots for expected length of parental leave separated by condition (Experiment 2). Diamonds represent means, horizontal lines represent medians.

Contrary to expectations, the presented prototypes of men did not substantially affect men’s *expected length of parental leave-taking*, *F*(3, 229) = 0.41, *p* = .747, η^2^ < .01, [.00; .02] (see Fig 4).

#### Exploratory analyses

**Fig 5:**
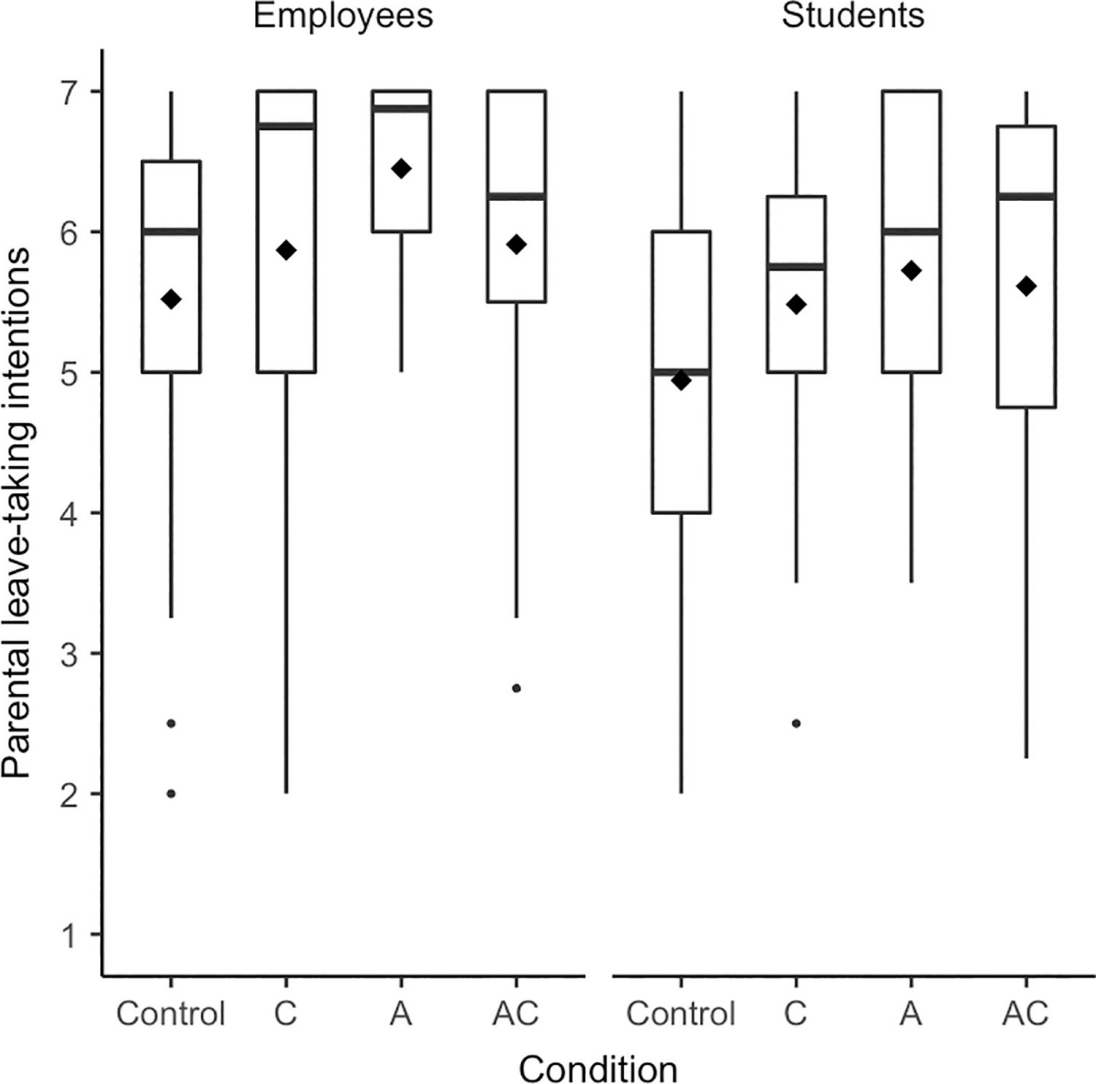
Boxplots for parental leave-taking intentions separated by condition and employment status (Experiment 2). (A) Employees. (B) Students. Diamonds represent means, horizontal lines represent medians.

The above results showed–in line with the central hypothesis (H1) and results from Experiment 1 –that men tended to report higher parental leave-taking intentions in the combined agentic and communal condition than in the control condition. Yet, in Experiment 2, men’s parental leave-taking intentions were even higher when the man of today was described as solely agentic. To better understand these findings, we ran exploratory analyses to see whether individual differences such as employment status or gender identification could help explain which condition is linked to more communal outcomes for whom. First, we compared the two biggest subsamples of Experiment 2: students and employees. As the sample in Experiment 1 and the samples in past research that we based our hypotheses on [74] only consisted of students, including employees was a unique feature of Experiment 2. We conducted an ANOVA with the factors condition (control vs. communion vs. agency vs. combination agency and communion) and employment status (employees vs. students; omitting the data of 23 participants with other employment status) and parental leave-taking intentions as the dependent variable (see Fig 5 for boxplots and S5 Text for details on statistical analyses). In addition to the main effect of condition, we found that in general employees had higher parental leave-taking intentions than students. Students, replicating Experiment 1, reported higher parental leave-taking intentions in the combined agentic and communal condition and now also in the agentic condition as compared to the control condition. In contrast, employees only reported higher parental leave-taking intentions in the agentic condition but not in any other condition as compared to the control condition.

Thus, although presenting an agentic prototype of men seems to have had stronger effects on men’s parental leave-taking intentions in Experiment 2 as compared to Experiment 1, students’ leave-taking intentions were in addition higher in the combined agentic and communal condition than in the control condition. Only for employees, contrast effects in the agentic condition prevailed. Hence, employment status (or being engaged in the agentic domain of work and breadwinning) seems to play a role for which composition of agency and communion in prototypes of men increases men’s intentions for communal engagement.

Besides employment status, we examined whether different degrees of gender identification, operationalized via a pictorial assessment of closeness between the self and the group of men [115, 116], played a role for which presented prototypes of men elicited self-reported communal outcomes in men. Thus, we conducted a moderation analysis including prototypes of men as the independent variable, gender identification as the moderator, and parental leave-taking intentions as the dependent variable (see S5 Text for details on statistical analyses). Degree of closeness between the self and the group of men significantly interacted with the combined agentic and communal condition. Probing the interaction revealed that the combined agentic and communal condition especially led to higher parental leave-taking intentions for men who did not feel very close or only moderately close to other men. These results suggest that the combined agentic and communal prototype of men may be particularly effective for men with little ties to the group of men, whereas the agentic prototype may be particularly effective for men who are already experienced in the male, agentic domain of work and breadwinning (provoking a contrast effect).

### Discussion

Experiment 2 showed only some support for the main hypothesis (H1) for parental leave-taking intentions. More specifically, being confronted with a prototypical representation of men that combined agency and communion tended to result in higher self-reported parental leave-taking intentions for men compared to the control condition. Moreover, the results for parental leave-taking intentions provided tentative support for H2: Presenting exclusively agentic prototypes of men also increased parental leave-taking intentions by trend. In line with H3, the communal prototype of men did not affect men’s communal outcomes. Yet, we also did not find substantial differences between the combined agentic and communal and the exclusively communal prototypes of men on any dependent variable, contradicting H4. In sum, the results of Experiment 2 were less clear than those of Experiment 1. Although we adapted the possible selves measures, these adaptations did not always yield stronger findings: Specifically, neither men’s communal possible self-concept nor communal possible task engagement were affected by degrees of agency and communion in prototypes of men (and neither was their expected length of parental leave). We discuss possible explanations for the mixed findings in the General Discussion.

## General discussion

Even though various benefits can result from men’s increased participation in communal roles, men remain underrepresented in traditionally female care-oriented engagement such as parental leave. Past research suggested that notions of what constitutes an ideal man or ideal father and the degree to which agentic and communal traits are integrated in these can play a crucial role for men’s orientation towards care. We thus examined to what extent suggesting different representations of their gender group affects men’s self-reported parental leave-taking intentions and possible selves with regard to work and care roles. We presented male participants with contrived newspaper articles on the man of today (control group: student or millennial of today) varying in agentic and communal content. Derived from the role prioritization model, we expected a prototypical representation of men that combines agentic and communal aspects to increase men’s communal intentions. The results of both experiments provided initial support for this main hypothesis for self-reported parental leave-taking intentions. Moreover, we found first evidence for contrast effects: As predicted, exclusively agentic prototypes of men were linked to higher parental leave-taking intentions. While the current results tend to support the main hypothesis that a combination of agency and communion in prototypical representations of men is likely to increase men’s parental leave-taking intentions, we found less support for effects on men’s possible selves and their expected length of parental leave. In addition to these mixed findings, two clear conclusions can be drawn from our research: First, both experiments showed that men expect to be highly engaged in communal roles in general. Second, consistent with our predictions and in contrast to what lay theories may expect, simply presenting communal prototypes of men to further promote men’s communal engagement clearly does not suffice.

### Assimilation and contrast effects and further mechanisms

In contrast to communal prototypes of men, we found that presentations of agentic prototypes were more likely to increase men’s parental leave-taking intentions. Whereas such a contrast effect is in line with hypotheses, we expected the combined agentic and communal condition to lead to participants reporting higher parental leave-taking intentions. Yet, this dominance of contrast effects is in line with findings from a meta-analysis of social comparison theory including assimilation and contrast effects. Especially in comparisons with actual persons, contrast effects seem to be the dominant response, and evidence for assimilation effects (i.e., being pulled towards a point of reference) is weak [117]. Still, we could argue that the increased communal intentions after being exposed to a more moderate and diverse prototype focusing on agency *and* communion could be interpreted as an assimilation effect: being pulled towards the newly added communal aspects of a prototype that combines traditional and emerging norms. Moreover, exploratory analyses of Experiment 2 showed that contrast effects were mainly driven by a specific subsample, employees, who were already engaged in the agentic domain of work and breadwinning. At the same time, parental leave represents a more realistic option for their immediate future because of their possibly higher age and career advancement, which is reflected in their overall higher parental leave-taking intentions as compared to students. Accordingly, being presented with a group prototype that only prescribes agentic engagement could lead to reactance [for reactance effects in a similar study see 75]. Alternatively, the agentic prototype of men could have functioned as a paradoxical intervention [118, 119]. Presenting participants with messages that are in line with their views but exaggerated can unfreeze their beliefs and lead to attitude change. In this case, the agentic prototypical representation of men could especially foster communal orientation in men with initially traditional gender-role attitudes. In contrast, communal intentions tended to be higher after being exposed to the combined agentic and communal prototype of men for other samples such as students, supporting findings from past research [74]. Moreover, in our Experiment 2, men who felt little to moderately close to their gender group reported higher parental leave-taking intentions following exposure to the combined agentic and communal prototype, suggesting that such prototypical descriptions of men could be especially effective for non-traditional men. In sum, whether a combination of agency and communion or an exclusive focus on agency in descriptions of what constitutes a man leads to more communal outcomes appears to depend on individual characteristics such as employment status and gender identification, and no clear conclusions can be drawn from our findings.

### Which prototypes should be effective for fostering men’s communal outcomes?

A further open question is why other communal outcomes such as men’s possible selves and the expected length of parental leave were less affected than parental leave-taking intentions. Results of a recent study that examined the role of professional prototypes for the underrepresentation of groups in professional contexts, such as women in firefighting, suggest that our manipulation could have lacked crucial aspects [75]. The authors propose that *balanced* category prototypes (the category being firefighting in this case), which emphasize both traits traditionally associated with the dominant group in this context as well as the non-dominant group, can reduce group-based biases and the underrepresentation of the non-dominant group, similar to our central hypothesis. However, their results [75] further showed that for participants to truly consider both groups of traits as equally important, it is necessary to especially emphasize the traits associated with the non-dominant group by presenting them first when ranked in order of importance (so-called prototype inversion). When looking at the category prototype of caretaking, this would mean that agency, which is traditionally associated with the non-dominant group of men in this context, should be emphasized. However, when considering group prototypes, as we did in the present research with prototypes of *men*, we could conclude that communion–traditionally associated with the female group–should be emphasized. In Experiment 1, we in fact included different orders of agency and communion. Yet, a reversed order in which communal attributes were mentioned first did not increase communal outcomes descriptively. However, an explanation for this could be that we did not stress that attributes mentioned first are more important for characterizing the group of men, which is what Danbold and Bendersky’s results would suggest. In addition to applying this prototype inversion, future research could consider other prototypes than prototypes of men such as, in the context of parental leave, the more specific group prototype of fathers. Moreover and as discussed, the results by Danbold and Bendersky pertain to category prototypes instead of group prototypes. As changing group prototypes has proven difficult in past research [for a discussion see 75], focusing on caretaking prototypes could be fruitful for motivating men to engage in such roles. In fact, men are already applying the strategy of redefining caretaking instead of redefining masculinity, for example, by defining childcare as “hard work” (i.e., as an agentic task) [52, 53]. Such an approach could especially lower barriers for highly identified men by reducing threats to their masculine identity. In addition, agency is associated with higher status and could thus contribute to increasing the appreciation of care work–a shift women could also benefit from. However, fully replacing or negating the traditional communal aspects of caretaking could have negative consequences for women who strongly identify with communion as well as for non-traditional communal men. These considerations are also outlined by Danbold and Bendersky [75] who therefore suggest focusing on balanced prototypes.

The idea of considering both aspects traditionally associated with gender groups, as well as those that have not, is also central to the role prioritization model on which we based the main hypothesis [73]. In the case of the present experiments, prototypical representations of men in which communal traits and behaviors complement agentic ones could reassure men and give them leeway to engage more in communal roles, as illustrated by their high parental leave-taking intentions in this condition (in addition to the agentic condition). Still, it remains unclear what is more effective for fostering counter-stereotypic outcomes: only adding communal aspects as an extra (augmentation) as Haines and Stroessner [73] suggest or specifically highlighting communal aspects as Danbold and Bendersky [75] suggest (yet it is additionally unclear to what extent the findings for category prototypes can be applied to group prototypes). Nevertheless, both, in addition to our work, stress the importance of integrating agency *and* communion to motivate counter-stereotypic engagement. This stands in contrast to what lay theories may assume: that focusing on the neglected aspects (communion in the case of men) will help to increase men’s communal orientation, for example regarding parental leave. One of the clearest findings of the present research is that presenting a communal prototype of men does not increase men’s self-reported intentions regarding parental leave and caretaking in general.

### Practice implications

The insight that communal prototypes are inefficient could, for example, be applied to gender portrayals in media and advertising. Past research showed that gender stereotyping is still prevalent in media and advertising cross-nationally [120–123]. For example, men are less often portrayed as engaging in domestic tasks and childcare than women, and if they are, these depictions are often characterized by lower competence and involvement as compared to women [124–126]. The ways in which men and male gender roles are portrayed in media and advertising is important, as media consumption has been linked to supporting and adhering to masculinity norms [127, 128]. Even though more non-traditional male portrayals focusing on caretaking and involved fatherhood are emerging, these often put a strong focus on communal, caretaking aspects while neglecting traditional male roles (e.g., the Dove Men+Care advertising line) [129]. The present research can be viewed as first evidence that communion-focused communication of masculinity norms could be ineffective in increasing men’s orientation towards care in contrast to communication including both communal and agentic aspects. Whereas increasing gender equality is not the (primary) goal of media content and advertising, this can be an important implication for societal and governmental communication that aims to reduce barriers to men’s engagement in communal roles.

Although some of our experimental conditions were more effective than others in fostering communal intentions, men reported generally high communal expectations. This finding is mirrored by representative population surveys in which 83% of young childless men think that fathers should spend as much time as possible with their children. Similarly, many fathers think that sharing childcare equally would be ideal. Yet only a minority actually does so [14], which indicates that men could be too optimistic about their involvement. In their model of cultural and psychological barriers to men’s engagement in communal roles, Croft and colleagues [1] propose that besides the internalization of communal traits, values, and possible selves, external barriers play a crucial role for men’s orientation towards care. Thus, even though men might be motivated, their engagement in childcare and parental leave further depends on, for example, financial costs and workplace or partner support [e.g., 22, 23, 25]. Future research could thus examine the (longitudinal) processes that seem to interfere with men’s initially high motivation, creating a gap between their intentions and behavior. Nevertheless, internal motivation is a necessary prerequisite for men to even consider increased communal engagement [except for cases in which policies create high incentives; 129, 130]. Even for men who are not confronted with childcare and leave-taking decisions themselves, valuing communion in men can lead them to support respective policies in organizations and societies. Such an increased support can, in turn, contribute to lowering external barriers which enable men to act on their internal motivations. In sum, findings of the present research imply that a communication of masculinity norms that is exclusively focused on communion is unlikely to foster men’s communal intentions. In fact, these communal intentions were high to begin with in the current samples, stressing the role of simultaneously lowering external barriers to men’s communal engagement and parental leave-taking by, for example, increasing social and financial support.

### Limitations

The findings of the present experiments should be viewed in light of several limitations. First, the samples of both experiments were skewed towards students who differ from general populations on fundamental psychological dimensions as shown by meta-analyses and reviews [107, 108]. Second, both experiments were conducted in Germany, and materials were partly tailored to the national policy context (i.e., the short information on parental leave policies in Germany presented to participants). As policies are an important driver of leave-taking decisions and vary considerably across countries [131–133], this constraint on the generality of the present results should be taken into account. In addition, we recruited male participants who indicated a desire to have children but were neither yet fathers nor expected to be so in the close future. Thus, it is possible that they had not yet fully developed attitudes towards parental leave-taking and therefore, especially in the case of their expected length of parental leave, gravitated towards a more or less egalitarian division of leave between themselves and their (future) partners [134, see also 135]. Nevertheless, we targeted these samples of highly educated childless men deliberately: Because of their own and their (future) partners’ possibly high education, financial considerations–otherwise an important external determinant of men’s leave-taking–could be less essential (and increasingly available financial compensation for paternal leave also makes this less key). For this group, it is therefore particularly important to understand how factors such as masculinity norms and ideas of what constitutes a man contribute to their behavioral intentions for communal engagement. At the same time, parental leave pay is capped at 1800€ in Germany. Although we informed participants about the national leave policy, information on the financial compensation that parents receive during parental leave was not included. Thus, for the targeted sample and considering gender norms continuously associating men with breadwinning, including this information could have affected men’s parental leave-taking intentions and reduced the observed ceiling effects.

Another limitation pertains to our chosen manipulation of prototypes. Theoretically, prototypes are context-dependent and can change according to the point of reference [61, 62]. However, the intergroup nature of prototypes was not directly reflected in the manipulation; thus, it can be argued whether we indeed manipulated prototypes. Still, we assume that the intergroup context of gender was activated in the present experiments due to the materials. As gender is traditionally viewed as binary (and this perception is only slowly changing) [136], it is likely that the group of women implicitly functioned as the point of reference. It is also an open question in which ways the prototypes’ category width or perceived distance to individuals affects men’s communal outcomes [e.g., 137, 138]. In the present research, we focused on prototypes of men in general; yet prototypes within men’s immediate environment (e.g., men in their profession or organization) could be more effective in drawing men towards communal engagement. However, men could also find it more difficult to distance themselves from an agentic male ideal if prototypes of men that are more closely related to their reality were described, possibly leading to lower communal intentions.

Finally, we are merely able to draw cautious conclusions based on the present findings. Only on some dependent variables (especially in Experiment 2), we found support for the main hypothesis that exposing male participants to a description of the man of today that includes agentic and communal content would increase men’s self-reported communal intentions. Even in Experiment 1 effects were smaller than initially expected, and the study may not have had enough power to clearly detect some effects. The results were further mixed across experiments and across dependent variables for the exclusively agentic prototypical representation of men that increased men’s self-reported communal intentions only sometimes. What is more, effects partly depended on outlier treatment and adjustment of error probabilities. We also did not find evidence for preregistered mechanisms behind these effects (e.g., assimilation and contrast effects or affirmation and threat responses, see Table 1 and S2 Text). Nevertheless, two clear findings emerged: We consistently found that men had generally high communal intentions and that a representation of men exclusively focused on communion does not further increase men’s orientation towards care.

## Conclusion

The current experiments offer first insight into how descriptions of what constitutes a man varying in agentic and communal content can generally affect men’s self-reported orientation towards care and their parental leave-taking intentions more specifically. We found initial evidence that a combination of agency and communion in presented prototypes of men can increase men’s parental leave-taking intentions, whereas an exclusive focus on agency additionally tends to foster leave-taking intentions in men via contrast or reactance effects. However, men’s possible selves and their broader orientation towards care were less affected. Except for the consistent finding that exclusively emphasizing communion in prototypical representations of men does not suffice to foster men’s communal intentions, we cannot draw clear conclusions based on the present findings. Further research is needed to clarify how men’s orientation towards care is affected by prototypical representations of their gender group and what the underlying mechanisms for these effects are. Generally though, men’s communal orientation was high to begin with, emphasizing the difficulty men have translating their communal orientation into actual communal behavior. An increased understanding of how men’s intentions for communal engagement are shaped by gendered norms enables us to develop ways to encourage their actual involvement and can ultimately contribute to gender-related social change.

## Supporting information

S1 Text Deviations from preregistration. (DOCX)

S2 Text Supplementary analyses. (DOCX)

S3 Text Changes in results based on inclusions. (DOCX)

S4 Text Additionally measured variables. (DOCX)

S5 Text Exploratory analyses for Experiment 2. (DOCX)
