# Supplementary material for: Which representations of their gender group affect men’s orientation towards care? the case of parental leave-taking intentions
Source: PLoS One. 2021 Dec 3;16(12):e0260950. doi: 10.1371/journal.pone.0260950 (PMC8641870; doi:10.1371/journal.pone.0260950)
Supplement: S1 Text — (DOCX) [file pone.0260950.s001.docx]

# Deviations from preregistration

In addition to the deviations outlined in the manuscript in Table 1, we preregistered to exclude participants who failed the manipulation check. Yet, this procedure is more advisable when the manipulation check is administered in the form of an attention check (e.g., whether participants remembered an important part of the manipulation correctly). In our case, it is not as clear what qualifies as a failed manipulation check, and excluding participants could invalidate the random assignment of participants to experimental conditions. Thus, we refrained from excluding cases based on this criterion.
