# Supplementary material for: Which representations of their gender group affect men’s orientation towards care? the case of parental leave-taking intentions
Source: PLoS One. 2021 Dec 3;16(12):e0260950. doi: 10.1371/journal.pone.0260950 (PMC8641870; doi:10.1371/journal.pone.0260950)
Supplement: S2 Text — (DOCX) [file pone.0260950.s002.docx]

# Supplementary analyses

## Experiment 1

### Perception of prototypes

We examined how the prototypical representations of men were perceived over and above agentic and communal attributions (for descriptive statistics see Table A). The prototypes differed as to how pleasant, *F*(3, 128) = 6.70, *p* < .001, $\eta$^2^ = .14, [.05; .22], extreme, *F*(3, 128) = 10.69, *p* < .001, $\eta$^2^ = .20, [.10; .29] and desirable, *F*(3, 128) = 3.30, *p* = .023, $\eta$^2^ = .07, [.01; .14], they were perceived. We found no substantial differences in how surprising they were perceived, *F*(3, 128) = 1.86, *p* = .139, $\eta$^2^ = .04, [.00; .10]. Especially perceptions of the prototypical representation of men focusing on agency differed from those of other prototypes. According to the participants, the description of the man of today in the agentic condition was more unpleasant than in the combined agentic and communal condition, *p* = .003, *d* = -0.84, [-1.34; -0.33], than in the communal condition, *p* = .001, *d* = -0.92, [-1.41; -0.44], and than in the control condition, *p* = .003, *d* = -0.90, [-1.41; -0.39]. The description of the man of today in the agentic condition was also perceived as more extreme than the description of the man of today in the combined agentic and communal condition, *p* < .001, *d* = 0.93, [0.42; 1.45], than that in the communal condition, *p* < .001, *d* = 1.07, [0.57; 1.56], and than that in the control condition, *p* < .001, *d* = 1.26, [0.73; 1.80]. Lastly, it was perceived as less desirable than the description of the man of today in the communal condition, *p* = .033, *d* = -0.69, [-1.16; -0.21]. The other conditions were not perceived substantially differently from each other, all *p*s > .055.

**Table A. Means and Standard Deviations for Perceptions of Prototypes in Experimental Conditions (Experiment 1).**

|  | **Experimental Condition** | | | |
| --- | --- | --- | --- | --- |
|  | *Control* | *Communion* | *Agency* | *Agency & Communion* |
| Unpleasant – pleasant | 64.37 (24.75) | 64.51 (23.86) | 42.00 (24.90) | 63.83 (27.47) |
| Unsurprising – surprising | 30.63 (22.76) | 43.68 (23.82) | 40.34 (26.56) | 34.77 (23.99) |
| Moderate – extreme | 37.80 (21.66) | 42.92 (20.44) | 66.31 (23.37) | 43.90 (24.84) |
| Undesirable – desirable | 56.97 (26.99) | 62.19 (23.71) | 45.20 (25.91) | 61.60 (25.68) |

Means with standard deviations in parentheses. Scales ranging from 0 to 100.

### Additional facets of possible selves and parental leave-taking

We conducted the same analyses of variance as in the manuscript for other facets of possible selves (i.e., desired and feared agentic and communal possible self-concept and possible task engagement) and other facets of parental leave-taking (i.e., desired [length of] leave-taking, perceived self-efficacy regarding leave-taking). We did not find any substantial differences between conditions on these variables, *F*s < 2.12, *p*s > .101, $\eta$^2^s < .05.

### Mediation analyses

#### As preregistered, we conducted mediation analyses to examine whether possible selves mediate the relation between prototypes of men and parental leave-taking variables. We did not find any significant indirect effects across operationalizations of possible selves and parental leave-taking, all bs < 0.45, $\beta$s < .07, all 95% Boot CIs included zero.

## Experiment 2

### Perception of prototypes

We again examined how the prototypical representations of men were perceived over and above agentic and communal attributions (for descriptive statistics see Table B). We expected the exclusively agentic prototype to be perceived as more extreme, unambiguous, and one-sided than the combined agentic and communal prototype (and vice versa; H2.5 in preregistration). In fact, the presented prototypes differed substantially regarding how extreme, *F*(3, 185) = 17.93, *p* < .001, $\eta$^2^ = .23, [.14; .30], unambiguous, *F*(3, 210) = 8.15, *p* < .001, $\eta$^2^ = .10, [.04; .17], and diverse they were perceived, *F*(3, 208) = 18.91, *p* < .001, $\eta$^2^ = .21, [.13; .29]. The agency-based prototypical description of men was perceived as more extreme than the combined agentic and communal prototype of men, *p* < .001, *d* = 1.28, [0.88; 1.68], than the communal prototype, *p* < .001, *d* = 1.36, [0.95; 1.78], and than that in the control condition, *p* < .001, *d* = 1.23, [0.83; 1.63]. The other conditions did not differ substantially from each other regarding perceptions of extremity, all *p*s > .750. Both the agentic and the communal prototypes of men were perceived as more unambiguous than the combined agentic and communal prototype (*p*_Avs.AC_ < .001, *d* = 0.77, [0.40; 1.15], *p*_Cvs.AC_ = .004, *d* = 0.67, [0.30; 1.04]) and than the control condition (*p*_Avs.Control_ < .001, *d* = 0.68, [0.30; 1.05], *p*_Cvs.Control_ = .004, *d* = 0.58, [0.21; 0.95]; all other *p*s > .584). In terms of diversity, the agentic prototype of men was perceived as less diverse (i.e., more one-sided) than the combined agentic and communal prototype, *p* < .001, *d* = -1.53, [-1.94; -1.11], than the communal prototype, *p* < .001, *d* = -1.05, [-1.45; -0.65], and than the control condition, *p* = .002, *d* = -0.63, [-1.00; -0.25]. Further, the combined agentic and communal prototype of men was perceived as more diverse than the control condition, *p* < .001, *d* = 0.76, [0.39; 1.13]. The communal prototype did not differ substantially from any other condition, all *p*s > .053. In sum, especially the agentic prototype of men was perceived as extreme, unambiguous, and one-sided and more so than the combined agentic and communal prototype, supporting H2.5. However, these different perceptions generally did not moderate the relation between prototypes of men differing in agentic and communal content and men’s communal outcomes (see below). Thus, we cannot conclude that assimilation and contrast are the mechanisms behind the obtained main effects of prototypes on, for example, parental leave-taking intentions.

**Table B. Means and Standard Deviations for Perceptions of Prototypes in Experimental Conditions (Experiment 2).**

|  | **Experimental Condition** | | | |
| --- | --- | --- | --- | --- |
|  | *Control* | *Communion* | *Agency* | *Agency & Communion* |
| Moderate – extreme | 4.61 (2.20) | 4.55 (1.95) | 7.11 (1.81) | 4.80 (1.81) |
| Ambiguous – unambiguous | 6.04 (2.58) | 7.37 (1.98) | 7.68 (2.24) | 6.02 (2.06) |
| One-sided – diverse | 5.16 (2.46) | 6.04 (2.30) | 3.71 (2.13) | 6.86 (2.00) |

Means with standard deviations in parentheses. Scales ranging from 1 to 10.

### Additional facets of possible selves, and suggested mechanisms

We conducted the same analyses of variance as in the manuscript for agentic possible selves and the second operationalization of possible task engagement. We did not find any substantial differences between presented prototypes of men on these variables, *F*s < 1.52, *p*s > .209, $\eta$^2^s < .02, except for the agentic possible self-concept, *F*(3, 229) = 3.99, *p* = .008, $\eta$^2^ = .05. Men tended to expect to be more agentic in the future in the communal as compared to the control condition, a contrast effect, *p* = .083, *d* = 0.35, [-0.01; 0.72].

We also examined whether the different prototypes of men affected whether men felt threatened or affirmed in their masculinity. We expected men to be more affirmed in their masculinity in the combined agentic and communal condition compared to the communal condition (and thus allowing for more communal outcomes). Moreover, we expected men to be more threatened in the communal condition compared to the control condition and compared to the combined agentic and communal condition. Contradicting these hypotheses (H2.4 in preregistration), we did not find any substantial differences between conditions, all *F*s < 1.08, *p*s > .358, $\eta$^2^ < .03. A possible explanation for these null results is the difficulty to measure threat (and possibly also affirmation) via self-report. Using self-report measures assumes that participants are aware of being threatened or affirmed in their masculine identity, although this cannot be taken as a given [1]. What is more, explicitly measuring threat responses has been linked to reactance and defensive behavior such as intentionally indicating low feelings of threat [see 2,3]. In fact, we obtained floor effects for threat measures; however, the data do not allow to distinguish whether these reflect reactivity or participants’ actual threat levels. Moreover, the masculine identity has been described as a precarious one, and thus self-presentation concerns could keep men from reporting that they feel threatened or affirmed in their masculinity [4,5]. Future research should therefore use physiological indices to assess affirmation and threat responses more comprehensively [2,6].

**Mediation and moderation analyses**

#### As preregistered, we conducted mediation analyses to examine whether possible selves as well as perceptions of threat and affirmation mediate the relation between prototypes of men and parental leave-taking variables. We did not find any significant indirect effects, all bs < 0.26, $\beta$s < .03, all 95% Boot CIs included zero.

We also examined whether self-typicality and the perceived extremity, ambiguity, and diversity of prototypes moderated the relations between prototypes of men and communal outcomes (i.e., communal possible selves and parental leave-taking variables). Self-typicality was operationalized via the self-stereotyping subscale of gender identification as well as Euclidean distances calculated separately for agency and communion between the perception of the presented prototype (as indicated on the manipulation check) and agentic and communal self-concepts. For the first operationalization of self-typicality, we found a significant interaction between the agentic and communal prototype of men and self-typicality, including parental leave-taking intentions as the dependent variable, *b* = -0.33, *SE* = 0.16, *t* = -2.06, *p* = .041 (overall *F*-Test: *F*(7, 225) = 2.63, *p* = .012, *R*^2^_adj_ = .05). Probing the interaction revealed that the combined agentic and communal condition especially led to higher parental leave-taking intentions for men who felt little like typical men (- 1 *SD*), *b* = 0.99, *SE* = 0.32, *t* = 3.07, *p* = .002, and also for men who felt moderately like typical men (*M*), *b* = 0.49, *SE* = 0.23, *t* = 2.15, *p* = .033.

Next, we conducted moderation analyses for the second operationalization of self-typicality. For self-typicality regarding communion, we found a significant interaction with the combined agentic and communal prototype of men, *b* = -0.59, *SE* = 0.15, *t* = -3.84, *p* < .001, and the communal prototype of men, *b* = -0.80, *SE* = 0.17, *t* = -4.76, *p* < .001, including the communal possible self-concept as the dependent variable (overall *F*-Test: *F*(7, 225) = 5.30, *p* < .001, *R*^2^_adj_ = .11). Probing the interactions indicated that the combined agentic and communal prototype of men led to more communal possible self-concepts for men who felt little like typical men regarding communion, *b* = 0.53, *SE* = 0.20, *t* = 2.61, *p* = .010. The same applies to the communal prototype of men, *b* = 0.62, *SE* = 0.20, *t* = 3.08, *p* = .002. Moreover, the combined agentic and communal, *b* = -0.67, *SE* = 0.22, *t* = -3.06, *p* = .002, as well as the communal prototype, *b* = -1.01, *SE* = 0.25, *t* = -3.98, *p* < .001, led to less communal possible self-concepts for men who felt much like typical men regarding communion.

When looking at the perceived diversity of prototypes as a moderator, we found a significant interaction with the combined agentic and communal prototype of men, *b* = -0.15, *SE* = 0.07, *t* = -2.03, *p* = .043, and the agentic prototype of men, *b* = -0.15, *SE* = 0.08, *t* = -2.06, *p* = .041, including the item “I will spend time looking after my children's needs” as the dependent variable (overall *F*-Test: *F*(7, 204) = 2.13, *p* = .042, *R*^2^_adj_ = .04). This item was part of the second operationalization of possible task engagement regarding childcare which did not form a reliable scale ($\alpha$ = .23). However, none of the conditional effects at levels of the moderator were significant (all *t*s < -1.86, *p*s > .064). For all other analyses, either the overall *F*-Test or the interactions were not significant. Thus, we did not find evidence for assimilation and contrast effects based on perceptions of prototypes as, for example, extreme, unambiguous, or one-sided (versus diverse).

## References

1. Blascovich J, Mendes WB. Challenge and threat appraisals: The role of affective cues. In: Forgas JP, editor. Feeling and thinking: The role of affect in social cognition. Cambridge (GB): Cambridge University Press; 2000. pp. 59–82.

2. Scheepers D, Ellemers N. When the pressure is up: The assessment of social identity threat in low and high status groups. J Exp Soc Psychol. 2005;41: 192–200. doi:10.1016/j.jesp.2004.06.002

3. Branscombe NR, Ellemers N, Spears R, Doosje B. The context and content of social identity threats. In: Ellemers N, Spears R, Doosje B, editors. Social identity: Context, commitment, content. Oxford (GB): Blackwell; 1999. pp. 35–58.

4. Vandello JA, Bosson JK, Cohen D, Burnaford RM, Weaver JR. Precarious manhood. J Pers Soc Psychol. 2008;95: 1325–1339. doi:10.1037/a0012453

5. Vandello JA, Bosson JK. Hard won and easily lost: A review and synthesis of theory and research on precarious manhood. Psychol Men Masc. 2012;14: 101–113. doi:10.1037/a0029826

6. Blascovich J, Tomaka J. The biopsychosocial model of arousal regulation. In: Zanna MP, editor. Advances in experimental social psychology. San Diego: Academic Press; 1996. pp. 1–51.
