# Supplementary material for: Which representations of their gender group affect men’s orientation towards care? the case of parental leave-taking intentions
Source: PLoS One. 2021 Dec 3;16(12):e0260950. doi: 10.1371/journal.pone.0260950 (PMC8641870; doi:10.1371/journal.pone.0260950)
Supplement: S3 Text — (DOCX) [file pone.0260950.s003.docx]

# Changes in results based on inclusions

## Experiment 1

As preregistered, we conducted all analyses again with cases that failed attention, suspicion, and quality checks. With these cases included, the overall *F*-test for communal possible task engagement was not significant, *F*(3, 140) = 2.36, *p* = .075, η^2^ = .05. Men now did not substantially expect communal tasks to be more typical for themselves in the future in the agentic condition as compared to the control condition, *p* = .064, *d* = 0.45, [-0.02; 0.92]. Similarly, the overall *F*-test for the expected length of parental leave-taking was not significant anymore, *F*(3, 140) = 1.99, *p* = .118, η^2^ = .04; yet the results for the comparisons between conditions did not change.

When outliers were included, participants did not substantially expect communal tasks to be more typical for themselves in the future in the agentic condition as compared to the control condition, *p* = .060, *d* = 0.40, [-0.06; 0.86]. Also, the overall *F*-test for the expected length of parental leave-taking was not significant anymore, *F*(3, 139) = 2.66, *p* = .051, η^2^ = .05. Although other findings did not change, men now only tended to expect to take longer leave in the combined agentic and communal condition as compared to the communal condition, *p* = .073, *d* = 0.53, [0.04; 1.01].

## Experiment 2

When we included cases that failed attention, suspicion, or quality checks, parental leave-taking intentions now only tended to differ between conditions, *F*(3, 235) = 2.57, *p* = .055, $\eta$^2^ = .03. In the exploratory 2x2 ANOVA including male prototypes and employment status as factors, students tended to have higher parental leave-taking intentions in the combined agentic and communion condition as compared to the control condition, *p* = .053, *d* = 0.47, [-0.04; 0.98], and in the agentic condition as compared to the control condition, *p* = .097, *d* = 0.42, [-0.09; 0.93] (all other *p*s > .110; both were significant when cases were excluded).

Including outliers did not affect our conclusions regarding hypotheses. Yet again, students only tended to have higher parental leave-taking intentions in the combined agentic and communal condition as compared to the control condition, *p* = .072, *d* = 0.47, [-0.04; 0.98] (all other *p*s > .150).
