# Supplementary material for: Which representations of their gender group affect men’s orientation towards care? the case of parental leave-taking intentions
Source: PLoS One. 2021 Dec 3;16(12):e0260950. doi: 10.1371/journal.pone.0260950 (PMC8641870; doi:10.1371/journal.pone.0260950)
Supplement: S4 Text — (DOCX) [file pone.0260950.s004.docx]

# Additionally measured variables

## Experiment 1

Regarding possible selves, we report results for expected possible selves in the manuscript but also assessed desired and feared possible selves, as well as the current self-concept together in a matrix. Similarly, we assessed desired parental leave-taking and desired length of parental leave in addition to perceived self-efficacy for parental leave with one item each. Moreover, participants were asked to indicate how comfortable they would feel telling their boss, their colleagues, and strangers that they take parental leave (/ of more than two months / more than their female partners) [adapted from 1]. We also assessed the general perception of the prototypes presented in the newspaper articles. Specifically, we asked participants to indicate their spontaneous impression of the given description of men (students) via five bipolar items: negative – positive (see manuscript), unpleasant – pleasant, unsurprising – surprising, not desirable – very desirable, moderate – extreme (all scales from 0 to 100). To measure how compatible participants consider agency and communion to be, we included the compatibility of communion, achievement and power scales (6 items) [2]. Moreover, we measured distinctiveness threat between men and women via three items adapted from Schmid, Hewstone, Tausch, Cairns, and Hughes [3]. We assessed the perceived complexity or diversity of men via six items adapted from Waldzus, Mummendey, Wenzel, and Weber [4] and Ehrke and Steffens [5]. Gender identification was measured by the centrality and self-stereotyping subscales from Roth and Mazziotta [6]. For the assessment of gender role attitudes, we used a measure from the German General Social Survey [7] consisting of ten items. Lastly, we created three items to measure the pressure to fulfill both agentic and communal roles (e.g., “The thought of having to be assertive and friendly at the same time stresses me”).

## Experiment 2

In addition to gender identification, operationalized via a pictorial assessment of closeness between the self and the group of men (see manuscript and exploratory analyses), we further assessed closeness between the self and the group of millennials as this was the prototype described in the control condition. After the manipulation, we again checked the perception of the presented prototypes as negative versus positive (see manuscript), moderate versus extreme, ambiguous versus unambiguous, and one-sided versus diverse (all scales from 1 to 10). As in Experiment 1, we assessed the current agentic and communal self-concept as well as agentic expected possible selves in addition to communal ones. For the agentic possible self-concept, one item from each of the four agentic subscales as described by Hentschel and colleagues [8] was used (e.g., effective, dominant). For the possible task engagement, we included items regarding non-routine household tasks and paid labor besides the ones reported in the manuscript [adapted from 9–12]. As a second operationalization, we asked participants to what extent six generated care-, housework-, and career-related statements will apply to them around the time when they want to have children (two items each, e.g., “I will spend time looking after all the needs of my children”).

To assess affirmation of masculine identity, we included the private subscale of the Collective Self-Esteem Scale [13] to measure state collective self-esteem (four items, e.g., “At this moment I feel good about being a man”) [14]. We assessed threat regarding the prototypicality of traditional men via nine generated items (e.g., “A modern image of masculinity threatens the status of men”). Furthermore, we assessed five threat-related emotions (e.g., nervous) [adapted from 14]. We also measured perceived self-typicality as a man via the self-stereotyping subscale of gender identification (three items) [6,15]. Lastly, we measured participants’ self-perceived care-giving competence at the time when they want to have children regarding a range of tasks (e.g., supervising morning routine) [adapted from 16] and included the Ambivalent Sexism Inventory [17,18] in a shortened version [following 19].

## References

1. van Grootel S, van Laar C, Meeussen L, Schmader T, Sczesny S. Uncovering pluralistic ignorance to change men’s communal self-descriptions, attitudes, and behavioral intentions. Front Psychol. 2018;9: 1–12. doi:10.3389/FPSYG.2018.01344

2. Block K, Schmader T. Perceived compatibility between communion and agency: A new construct and measure. 2016.

3. Schmid K, Hewstone M, Tausch N, Cairns E, Hughes J. Antecedents and consequences of social identity complexity: Intergroup contact, distinctiveness threat, and outgroup attitudes. Pers Soc Psychol Bull. 2009;35: 1085–1098. doi:10.1177/0146167209337037

4. Waldzus S, Mummendey A, Wenzel M, Weber U. Towards tolerance: Representations of superordinate categories and perceived ingroup prototypicality. J Exp Soc Psychol. 2003;39: 31–47. doi:10.1016/S0022-1031(02)00507-3

5. Ehrke F, Steffens MC. Not so typical after all: Self-typicality moderates majority members’ reactions to diversity and attitudes towards minorities. Manuscript in preparation; 2018.

6. Roth J, Mazziotta A. Adaptation and validation of a German multidimensional and multicomponent measure of social identification. Soc Psychol (Gott). 2015;46: 277–290. doi:10.1027/1864-9335/a000243

7. Walter JG. Measures of gender role attitudes under revision: The example of the German General Social Survey. Soc Sci Res. 2018;72: 170–182. doi:10.1016/j.ssresearch.2018.02.009

8. Hentschel T, Heilman ME, Peus C v. The multiple dimensions of gender stereotypes: A current look at men’s and women’s characterizations of others and themselves. Front Psychol. 2019;10: 1–19. doi:10.3389/fpsyg.2019.00011

9. Craig L, Powell A. Shares of housework between mothers, fathers and young people: Routine and non-routine housework, doing housework for oneself and others. Soc Indic Res. 2018;136: 269–281. doi:10.1007/s11205-016-1539-3

10. Croft A, Schmader T, Block K. Life in the balance: Are women’s possible selves constrained by men’s domestic involvement? Pers Soc Psychol Bull. 2019;45: 808–823. doi:10.1177/0146167218797294

11. Endendijk JJ, Derks B, Mesman J. Does parenthood change implicit gender-role stereotypes and behaviors? J Marriage Fam. 2018;80: 61–79. doi:10.1111/jomf.12451

12. Yavorsky JE, Kamp Dush CM, Schoppe-Sullivan SJ. The production of inequality: The gender division of labor across the transition to parenthood. J Marriage Fam. 2015;77: 662–679. doi:10.1111/jomf.12189

13. Luhtanen R, Crocker J. A collective self-esteem scale: Self-evaluation of one’s social identity. Pers Soc Psychol Bull. 1992;18: 302–318.

14. Derks B, Van Laar C, Ellemers N. Working for the self or working for the group: How self- versus group affirmation affects collective behavior in low-status groups. J Pers Soc Psychol. 2009;96: 183–202. doi:10.1037/a0013068

15. Leach CW, van Zomeren M, Zebel S, Vliek MLW, Pennekamp SF, Doosje B, et al. Group-level self-definition and self-investment: A hierarchical (multicomponent) model of in-group identification. J Pers Soc Psychol. 2008;95: 144–165. doi:10.1037/0022-3514.95.1.144

16. Barnett RC, Baruch GK. Determinants of fathers’ participation in family work. J Marriage Fam. 1987;49: 29. doi:10.2307/352667

17. Eckes T, Six-Materna I. Hostilität und Benevolenz: Eine Skala zur Erfassung des ambivalenten Sexismus. Zeitschrift für Sozialpsychologie. 1999;30: 211–228. doi:10.1024/0044-3514.30.4.211

18. Glick P, Fiske ST. The Ambivalent Sexism Inventory: Differentiating hostile and benevolent sexism. J Pers Soc Psychol. 1996;70: 491–512. doi:10.1037/0022-3514.70.3.491

19. Sibley CG, Becker JC. On the nature of sexist ambivalence: Profiling ambivalent and univalent sexists. Eur J Soc Psychol. 2012;42: 589–601. doi:10.1002/ejsp.1870
