# Supplementary material for: Which representations of their gender group affect men’s orientation towards care? the case of parental leave-taking intentions
Source: PLoS One. 2021 Dec 3;16(12):e0260950. doi: 10.1371/journal.pone.0260950 (PMC8641870; doi:10.1371/journal.pone.0260950)
Supplement: S5 Text — (DOCX) [file pone.0260950.s005.docx]

# Exploratory analyses for Experiment 2

To better understand the findings of Experiment 2, we ran exploratory analyses to see whether individual differences such as employment status or gender identification could help explain which condition leads to more communal outcomes for whom. First, we compared the two biggest subsamples of Experiment 2: students and employees. We conducted an ANOVA with the factors condition (control vs. communion vs. agency vs. combination agency and communion) and employment status (employees vs. students; omitting the data of 23 participants with other employment status) and parental leave-taking intentions as the dependent variable. Specifically, we found – in addition to the main effect of condition, *F*(3, 202) = 3.89, *p* = .010, $\eta_{p}$^2^ = .05 – a significant main effect of employment status, *F*(1, 202) = 7.62, *p* = .006, $\eta_{p}$^2^ = .04. Employees had higher parental leave-taking intentions than students in general. Students, replicating Experiment 1, had higher parental leave-taking intentions in the combined agentic and communal condition, *p* = .045, *d* = 0.47, [-0.04; 0.98]. In addition, students also had higher parental leave-taking intention in the agentic condition, *p* = .022, *d* = 0.61, [0.09; 1.13], as compared to the control condition (all other *p*s > .111). In contrast, employees only had higher parental leave-taking intentions in the agentic condition but not in any other condition as compared to the control condition, *p* = .015, *d* = 0.80, [0.19; 1.41],

Besides employment status, we examined whether different degrees of gender identification (*M* = 4.63, *SD* = 1.68), operationalized via a pictorial assessment of closeness between the self and the group of men [1,2], played a role for which degrees of agency and communion in prototypes of men elicited communal outcomes in men. Thus, we conducted a moderation analysis including prototypes of men as the independent variable, gender identification as the moderator, and parental leave-taking intentions as the dependent variable, *F*(7, 225) = 2.44, *p* = .020, *R*^2^_adj_ = .04. Degree of closeness between the self and the group of men significantly interacted with the combined agentic and communal condition, *b* = -0.36, *SE* = 0.14, *t* = -2.51, *p* = .013. Probing the interaction revealed that the combined agentic and communal condition especially led to higher parental leave-taking intentions for men who did not feel very close to other men (-1 *SD*), *b* = 1.09, *SE* = 0.32, *t* = 3.41, *p* = .001, and also for men who felt moderately close to other men (*M*), *b* = 0.49, *SE* = 0.23, *t* = 2.13, *p* = .034.

## References

1. Schubert TW, Otten S. Overlap of self, ingroup, and outgroup: Pictorial measures of self-categorization. Self Identity. 2002;1: 353–377. doi:10.1080/152988602760328012

2. Tropp LR, Wright SC. Ingroup identification as the inclusion of ingroup in the self. Pers Soc Psychol Bull. 2001;27: 585–600. doi:10.1177/0146167201275007
